# Comparative efficacy and safety of Chinese medicine injections combined with capecitabine and oxaliplatin chemotherapies in treatment of colorectal cancer: A bayesian network meta-analysis

**DOI:** 10.3389/fphar.2022.1004259

**Published:** 2022-11-29

**Authors:** Shuzhen Liu, Kun Zhang, Xianfang Hu

**Affiliations:** ^1^ Shandong University of Traditional Chinese Medicine, Jinan, China; ^2^ Linyi Traditional Chinese Medicine Hospital, Linyi, China

**Keywords:** Chinese herbal injections, XELOX regimen, network meta-analysis, colorectal cancer, randomized controlled trials

## Abstract

**Objective:** The aim of the present Bayesian network meta-analysis (NMA) was to explore the comparative effectiveness and safeaty of different Chinese Medicine injections (CMIs) combined with the XELOX regimen *versus* XELOX alone for colorectal cancer (CRC).

**Methods:** A comprehensive search for randomized controlled trials (RCTs) was performed with regard to different CMIs for the treatment of CRC in several electronic databases up to April 2022. The quality assessment of the included RCTs was conducted according to the Cochrane risk of bias tool. Standard pair-wise and Bayesian NMA were designed to comparethe effectiveness and safety of different CMIs combined with the XELOX regimen by utilizing R 4.0.3 software and Stata 15.1 software simultaneously.

**Results:** Initially, a total of 4296 citations were retrieved through comprehensive searching, and 32 eligible articles involving 2847 participants and 11 CMIs were ultimately included. CMIs combined with XELOX were superior to the XELOX regimen alone, and a total of ten Observation Indicators were included in the study, with the following results. Among all the injections, Shengmaiyin, Shenmai, and Kanglaite combined with the XELOX regimen were the three CMIs with the highest clinical efficiency. The top three in terms of improving CD3^+^ values were Shengmaiyin, Shenqifuzheng, and Cinobufacini injections. Shenqifuzheng, Shengmaiyin, and BruceaJavanica oil injections combined with the XELOX regimen performed best at raising CD4^+^ values. Kanglaite, Cinobufacini, and Matrine injections combined with the XELOX regimen performed best in improving CD4+/CD8+ rates. The top three in terms of improving performance status were Xiaoaiping, Shenmai, and Kanglaite injections. Cinobufacini and Brucea Javanica oil injections combined with the XELOX regimen performed best at raising CD8^+^ values. Shenqifuzheng, Kangai, and Matrine injections combined with the XELOX regimen performed best in improving Gastrointestinal reactions.The top threein terms of improving Leukopenia were Shenqifuzheng, Compound Kushen and Kanglaite injections. The top three in terms of improving Platelet decline were Compound Kushen, Cinobufacini and Shenqifuzheng injections. Additionally, those that were best at improving nausea and vomitting were Cinobufacini, Compound Kushen and Aidi injections.

**Conclusion:** The results of the analysis demonstrated thatShengmaiyin, Kanglaite, and Cinobufacini injections and the XELOX regimen were associated with morepreferable and beneficial outcomes than other CMI groups. Nevertheless, additional results from multicenter trials and high-quality studies will bevital to support our findings.

**Systematic Review Registration:** https://www.crd.york.ac.uk/PROSPERO/display_record.php?RecordID=326097, CRD42022326097.

## Introduction

Colorectal cancer is the third most common cancer in the world and the second leading cause of cancer-related death (Chen et al., 2018). In China, the incidence and mortality rate of colorectal cancer are on the rise, and colorectal cancer ranks among the five most common malignant tumors in all regions. CRC is also a common cause of death due to a malignant tumor in all regions (Chen et al., 2014; Sun et al., 2015). The epidemiology of cancer varies among different regions and within different age, sex, and racial groups. This variation involves a variety of factors, including risk factor exposure, demographic changes, genetic susceptibility, etc. Nationwide, the incidence of CRC is decreasing at a rate of 2% per year. However, there is a progressive trend toward a younger incidence of CRC, with the incidence of cancer in patients under 50 years of age increasing (Zhang and Zhao, 2000; Weinberg et al., 2017; Henry and Johnson, 2019).

Currently, the main treatment for colorectal cancer is surgery. Five-year survival for CRC was calculated as 46.8% and 48.4% for men and women, respectively, in a composite estimate of 51 registries in 23 countries, and approximately 30% of patients will experience recurrence after surgery (Coleman et al., 2008; Labianca et al., 2013). In addition, colorectal cancer patients are burdened by longer treatment cycles, more expensive treatment, and more adverse effects. In the 2022 NCCN update of the colorectal cancer guidelines, the XELOX regimen was recommended as Class 1 adjuvant chemotherapy for stage III colon cancer. Moreover, XELOX is also recommended for stage II colon cancer with a high risk of systemic recurrence. In terms of five-year overall survival figures, the three-month XELOX adjuvant regimen is less toxic than the six-month regimen (Andre et al., 2004; Saltz et al., 2008; Andre et al., 2009; André et al., 2020). Studies have also demonstrated that the XELOX regimen can improve disease-free survival (DFS) in patients with stage III colon cancer, and that this benefit is long-lasting (Haller et al., 2011).

Chemotherapy drugs cause more damage to patients themselves, with a higher incidence of adverse reactions such as leukopenia, liver and kidney function damage, and vomiting. CMIs are guided by the theory of traditional Chinese medicine, using modern science and technology, produced from the effective substances extracted from the single or compound prescriptions of traditional Chinese medicine and natural drugs. The effective drug concentration of Chinese medicine injection is high, and it is easy to apply and shows faster efficacy; thus, it is widely used in clinical practice (Jiang et al., 2014). Anti-cancer medicine injection is mainly used as an adjuvant radiotherapy and chemotherapy for tumors, with the functions of reducing toxicity, improving symptoms, and enhancing therapeutic efficacy, etc. (Cheng and Liu, 2009; Zhao et al., 2016). Numerous clinical studies have found that Chinese medicine treatment of tumors, especially the application of Chinese medicine after radiotherapy and chemotherapy, can not only improve the therapeutic effect but also alleviate the adverse effects caused by radiotherapy and chemotherapy; therefore, Chinese medicine is widely used in the comprehensive treatment of tumors (Luo et al., 2010). In Zhang’s study (Zhang et al., 2017), the meta analysis of CMIs were compared and concluded that Compound Kushen, Kangai or Kanglaite injection combined with chemotherapy yielded significantly higher probability of improving performance status for patients with pancreatic cancer. In Wang‘s study (Wang et al., 2014), the meta analysis of CMIs were compared and concluded that Kanglaite, *Astragalus* polysaccharides or Brucea Javanica Oil combined with FOLFOX had the greatest probability of being the best treatment in clinical efficacy and safety for patients with gastric cancer. Therefore, by comparing the meta analysis of CMIs and concluding that CMIs are the better treatment in clinical efficacy and safety is desirable.

Network meta-analysis (NMA) can be used to synthesize multiple correlation factors and perform direct or indirect comparisons simultaneously by summarizing different interventions for the same disease. Moreover, NMA can provide evidence for the identification of optimal therapies based on the rankings of different outcomes. Given the widespread and long-term use of CMIs combined with chemotherapy in China, it is necessary to explore the comparative effectiveness and safety of different CMIs plus XELOX against CRC. To address this issue, this NMA was conducted to provide reference points regarding the clinical incorporation of CMIs as adjuvant chemotherapy for CRC.

## Materials and methods

This study was conducted following the PRISMA extension statement(Hutton et al., 2015) with a PRISMA checklist which is provided in Supplementary File S1.

### Search strategy

We searched relevant databases including PubMed, Embase, Cochrane Library, Web of Science, China National Knowledge Infrastructure Database (CNKI), Wan-Fang Database, Chinese Scientific Journals Full-text Database (VIP), and the Chinese Biomedical Literature Database (SinoMed) inception to 1 April 2022. The main searched terms related to “Colorectal Neoplasm”,“Colonic Neoplasm”, “Rectal Neoplasm”, “Injection”, “randomized controlled trial”. The search strategies are provided in Supplementary File S2.

### Inclusion criteria

#### Types of studies

Randomized controlled trials (RCTs) regarding CMIs combined with XELOX in the treatment of CRC were eligible, which is referred to as “random”, with or without blinding.

#### Types of participants

All patients were diagnosed with CRC pathologically and histologically, no limitation on gender and nationality.

#### Types of interventions

Patients in control group only received XELOX chemotherapy regimens, including Capecitabine and Oxaliplatin. Patients in treatment group received CMIs with XELOX therapy.

#### Types of outcomes

Primary outcomes include clinical effectiveness rate, performance status, T-lymphocyte subsets (including CD3^+^, CD4^+^, CD8^+^,CD4+/CD8+), Gastrointestinal reactions, nausea and vomitting, Leukopenia and Platelet decline. According to the WHO Objective Response Criteria in Solid Tumors, The clinical effectiveness rate [numberof complete response (CR) patients + partial response (PR)]/total number of patients ×100%. Performance status is the Karnofsky Performance Status(KPS), in accordance with KPS functional status scoring criteria, there are three levels: improvement (KPS score increased by more than 10 points), stability (KPS score changed by less than 10 points) and decrease (KPS score decreased by more than 10 points). An increase of more than 10 points in KPS score is considered as a significant improvement in performance status. RCTs that haveat least any one of the primary outcome indexes were included in this study.

### Exclusion criteria

The exclusion criteria were as follows: 1) For the repeatedly published articles, only remained the latest or more comprehensive ones; 2) Excluding meta-analysis, retrospective studies, case reports, animal experiments, conference summary, guide, index, non-RCT, non-English and Chinese papers; 3) Excluding intervention that does not meet the requirements or disease that does not match; 4) The article could not be obtained; 5) No mention of chemotherapy regimen or chemotherapy regimen does not match.

### Selection criteria

Two researchers independently screened the literature according to the inclusion criteria and exclusion criteria based on PICOS.

### Data extraction and quality assessment

Data regarding trial information (first-author, publication year, sample size, tumor stage, trial duration, interventions, and control), population characteristics (age and sex), reported outcomes (the clinical effectiveness rate, performance status, CD3^+^, CD4^+^, CD8^+^, CD4+/CD8+, gastrointestinal reactions, nausea and vomitting, leukopenia, platelet decline), information on methodology (blinding, random methods, and measurement of each indicator), were extracted by two independent reviewers using Excel software.Two investigators (KZ and SZL) independently assessed the quality of all eligible studies using the Cochrane Collaboration’s Risk of Bias tool (Higgins et al., 2011) to rate each item criterion of studies as either at low risk of bias, unclear risk of bias, and high risk of bias, across the following seven domains: random sequence generation, allocation concealment, blinding of participants and personnel, blinding of outcome assessment, incomplete outcome data, selective outcome reporting, and other bias. Any disagreements in the risk of bias assessment were resolved and evaluated by a discussion with a third investigator (XFH).

### Statistical analysis

The clinical effectiveness rate, Gastrointestinal reactions, nausea and vomitting, Leukopenia and Platelet decline were displayed as a risk ratio (RR) with 95% confidence intervals (CIs). The performance status, CD3^+^, CD4^+^, CD8^+^, and the rate of CD4+/CD8+ were displayed as weighted mean differences (MD) with 95% CIs. In view of the heterogeneity between trials, the Bayesian hierarchical random-effects model was first fitted for multiple comparisons of different treatment options for CRC (Dias et al., 2013; Mills et al., 2013). On the one hand, all the calculations and graphs were obtained using the R 4.0.3 software and Stata 15.1 software. Based on the theory of likelihood function and some prior assumptions, Markov chain Monte Carlo (MCMC) simulation was performed using Bayesian inference with R 4.0.3 software, 500,000 in iterations and 20,000 in annealing were set, to investigate the posterior distributions of the interrogated nodes (Dias et al., 2012; Bois, 2013; Hamra et al., 2013). On the other hand, the relationships among the different treatments were presented as a network graph; meanwhile, a comparison-adjusted funnel plot was utilized to test for potential publication bias (Chaimani et al., 2013; Youdom et al., 2017). Moreover, we adopted surface under the cumulative ranking probabilities (SUCRA) values to rank the examined treatments, and the SUCRA values ranged from 0 to 1. A higher SUCRA value corresponds to a higher ranking for CRC compared with other treatments (Rücker and Schwarzer, 2015; Trinquart et al., 2016). A league table was generated to present the comparisons between each pair of interventions within each outcome. Because there was no head-to-head trial in the NMA, the consistency assumption was not established (White et al., 2012). Furthermore, sensitivity analyses were conducted to assess the robustness of the results and deal with heterogeneity. The risk of bias was generated by RevMan (version 5.4) for all included studies. Cluster analysis based on the SUCRA values of the selected CMIs + XELOX within each outcome (the clinical effectiveness rate, CD3^+^, CD4^+^, CD8^+^, CD4+/CD8+, performance status, gastrointestinal reactions, leukopenia, platelet decline, nausea and vomitting) was performed.

## Results

### Literature and assessment of quality

**FIGURE 1:**
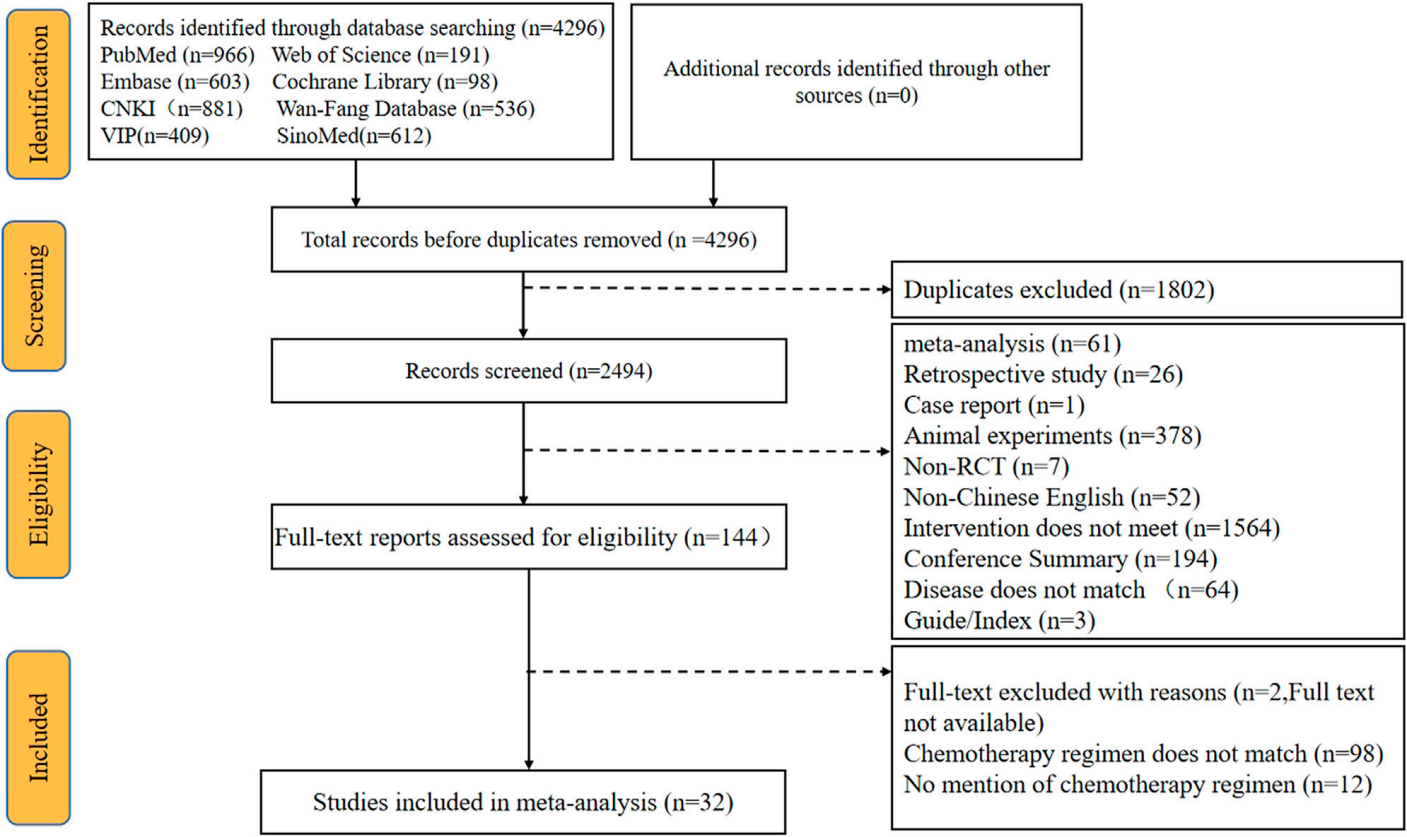
Flow chart of the search for eligible studies.

A total of 4296 articles were retrieved *via* the searching of the literature databases (see Materials and Methods). After screening the titles and abstracts to remove irrelevant articles and reading the full texts to eliminate articles that did not meet the inclusion criteria, ultimately, a total of 32 RCTs that evaluated CMIs combined with the XELOX regimen for the treatment of CRC were identified. In addition, this NMA incorporated 11 types of CMIs, namely, Shengmaiyin, Shenmai, Kanglaite, Shenqifuzheng, Cinobufacini, Brucea Javanica Oil, Matrine, Xiaoaiping, Aidi, Kangai, and Compound Kushen injections (see Supplementary Table S1 for characteristics of the included CMIs). All trials were published in Chinese, and the flow diagram is presented in Figure 1.

**FIGURE 2:**
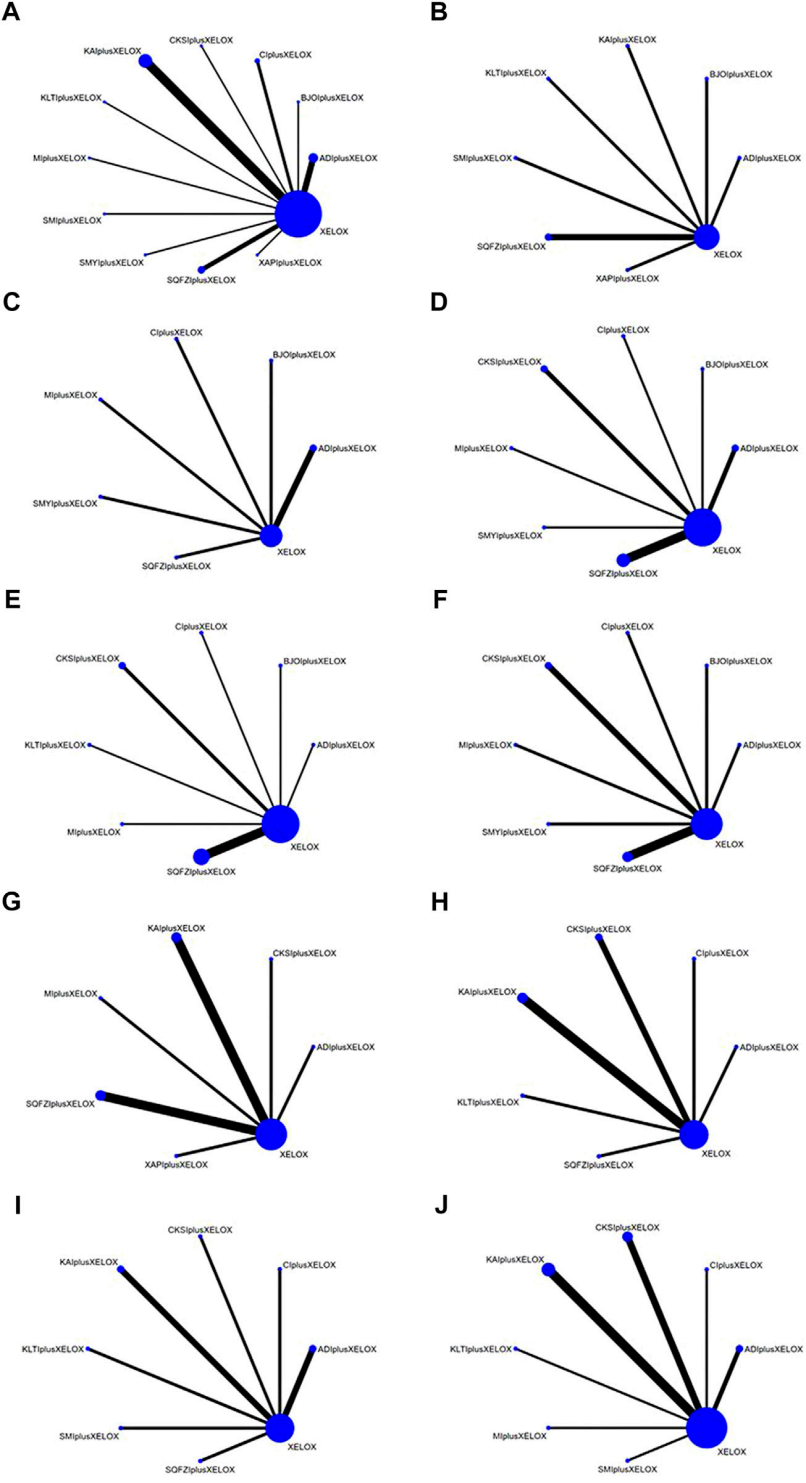
Network graphs of outcomes: **(A)** the clinical effectiveness rate; **(B)** performance status; **(C)** CD3^+^; **(D)** CD4^+^; **(E)** CD4+/CD8+; **(F)** CD8^+^; **(G)** Gastrointestinal reactions; **(H)** Leukopenia; **(I)** Platelet decline; **(J)** nausea and vomitting.

Overall, 2847 patients with CRC from 32 RCTs were involved in the present NMA; among them, 1435 patients were allocated to CMIs–XELOX, and 1412 patients received XELOX alone (Shi et al., 2009; Ling and Zhao, 2011; Tan et al., 2013; Du, 2014; Ruan et al., 2014; Shi and Dong, 2014; Wu, 2014; Kong and Xie, 2015; Miao and Gong, 2015; Wang, 2015; Zhang et al., 2015; Ren et al., 2016; Wang and Xu, 2016; Ding et al., 2017; Gu and Li, 2017; Xu, 2017; Wu et al., 2018; Zhang, 2018; Guo et al., 2019; Ming and Zou, 2019; Zhang and Zhang, 2019; Fu and Zhang, 2020; Liu, 2020; Pan, 2020; Yin et al., 2020; Zhou and Li, 2020; Chai et al., 2021; Li, 2021; Tian, 2021; Wang et al., 2021; Li; Zhang and Hou, 2021). All of the included RCTs reported information on patient population sizes, sex, and age; except for 52 and 60 trials where the tumor stage of the patients was not described, all patients in the trials had advanced tumors, while 31, 35, 41, 49, 51, 52, and 61 trials described the patients’ disease duration. There was no major difference in patient characteristics between different treatment arms. The basic characteristics of the individual trials are listed in Table 1 and Figure 2 shows the network graph of different interventions for the outcomes.

**TABLE 1:** The basic characteristics of the included RCTs.

	N(E/C)	Average Age	Sex(M/F)	Pathological type		Disease duration	Intervention	Control group measure	Treatment(days)	Outcomes
Study ID				Early	Advanced					
Fu et al.2020^32^	E:52	E:60.39 ± 7.20	E:30/22		√	E:(2.96 ± 0.75)a	ADI 50-100 ml + XELOX	XELOX	14d × 2	①⑥⑦⑨
	C:52	C:60.08 ± 7.12	C:29/23			C:(3.02 ± 0.81)a				
Shi et al.2009^33^	E:18	36–78	19/17		√	NR	ADI 50-100 ml + XELOX	XELOX	>14d × 2	①⑧⑨⑩
	C:18									
Wu et al.2018^34^	E:43	E:50.1 ± 5.9	E:28/15		√	NR	ADI 100 ml + XELOX	XELOX	21d × 3	①②③④⑤⑩
	C:43	C:49.8 ± 6.2	C:30/13							
Chai et al.2021^35^	E:149	E:61.33 ± 12.27	E:83/66		√	NR	CKSI 12 ml + XELOX	XELOX	7d × 4	⑧⑨⑩
	C:149	C:59.37 ± 10.28	C:78/71							
Zhou et al.2020^36^	E:45	E:52.6 ± 8.1	E:26/19		√	E:(8.2 ± 2.0)m	ADI 50 ml + XELOX	XELOX	42d	①
	C:45	C:53.1 ± 8.4	C:28/17			C:(8.4 ± 2.1)m				
Zhang et al.2015^37^	E:43	E:63.5 ± 6.7	E:28/15		√	NR	SQFZI 250 ml + XELOX	XELOX	14d × 2	①③④⑤⑥
	C:43	C:64.3 ± 7.2	C:29/14							
Tan et al.2013^38^	E:20	52–72	28/12		√	NR	SQFZI 250 ml + XELOX	XELOX	≥14d × 2	①③④⑤⑥
	C:20									
Zhang et al.2021^39^	E:43	E:71.89 ± 6.47	E:25/18		√	NR	SQFZI 250 ml + XELOX	XELOX	21d × 4	①②⑤⑦⑧⑨
	C:43	C:72.35 ± 5.36	C:27/16							
Li.2021^40^	E:40	E:53.4 ± 12.3	E:26/14		√	NR	CKSI 15 ml + XELOX	XELOX	10d × 4	③④⑤⑧⑩
	C:40	C:53.5 ± 12.2	C:28/12							
Yin et al.2020^41^	E:68	E:53.9 ± 4.0	E:35/33		√	NR	CKSI 12 ml + XELOX	XELOX	21d × 4	③④⑤
	C:68	C:54.2 ± 3.5	C:38/30							
Zhang et al.2019^42^	E:40	E:55.15 ± 10.16	E:25/15		√	E:7m-8a	SMI 100 ml + XELOX	XELOX	10d	①⑥⑨⑩
	C:40	C:53.57 ± 10.23	C:24/16			C:8m-7a				
Xu.2017^43^	E:23	E:56.45 ± 9.21	E:15/8		√	NR	SQFZI 250 ml + XELOX	XELOX	14d × 3	⑦
	C:23	C:57.45 ± 9.09	C:16/7							
Guo et al.2019^44^	E:75	60–75	E:45/30		√	NR	KAI 60 ml + XELOX	XELOX	14d × 2	①⑦
	C:73		C:52/21							
Ruan et al.2014^45^	E:34	E:35–63	E:18/16		√	NR	KAI 40 ml + XELOX	XELOX	>14d × 2	①⑧⑨⑩
	C:33	C:34–67	C:15/18							
Wang et al.2021^46^	E:34	E:54.30 ± 8.27	E:21/13		√	NR	KAI 40 ml + XELOX	XELOX	14d × 2	①⑩
	C:34	C:53.26 ± 8.41	C:20/14							
Li.2015^47^	E:48	E:56.72 ± 7.24	E:27/21		√	NR	KAI 40 ml + XELOX	XELOX	14d × 3	①
	C:45	C:57.13 ± 7.05	C:25/20							
Ding et al.2017^48^	E:32	E:35–76	E:20/12		√	NR	KAI 40 ml + XELOX	XELOX	14d × 2	①⑦
	C:30	C:37–78	C:18/12							
Ling et al.2011^49^	E:33	60–76	38/28		√	NR	KLTI 100 ml + XELOX	XELOX	14d × 2	①⑤⑥⑧⑨⑩
	C:33									
Ren et al.2016^50^	E:40	E:55.7 ± 11.2	E:25/15		√	E:(6.5 ± 2.4)m	MI 150 mg + XELOX	XELOX	14d × 6	①②③④⑤⑦⑩
	C:40	C:56.2 ± 10.4	C:28/12			C:(6.3 ± 2.2)m				
Zhang.2018^51^	E:40	E:62.24 ± 2.68	E:21/19	NR	NR	SMYI 60 ml + XELOX	XELOX	14d × 8	①②③④	
	C:40	C:63.51 ± 24.21	C:22/18							
Pan.2020^52^	E:35	E:60.2 ± 2.3	E:21/14		√	E:(7.1 ± 1.2)a	CI 15-20 ml + XELOX	XELOX	7d × 6	①⑨⑩
	C:35	C:59.8 ± 2.5	C:20/15			C:(7.3 ± 1.1)a				
Tian.2021^53^	E:40	E:59.59 ± 6.09	E:23/17		√	E:(1.95 ± 0.35)a	ADI 50 ml + XELOX	XELOX	21d × 3	②③
	C:40	C:58.49 ± 5.83	C:25/15			C:(1.85 ± 0.37)a				
Ming et al.2019^54^	E:48	E:54.77 ± 10.09	E:31/17		√	NR	BJOI 30 ml + XELOX	XELOX	21d × 3	①②③④⑤⑥
	C:48	C:54.78 ± 10.12	C:30/18							
Miao et al.2015^55^	E:29	E:59.4 ± 8.0	E:14/15		√	NR	SQFZI 250 ml + XELOX	XELOX	21d × 3	③⑤
	C:29	C:58.9 ± 8.2	C:15/14							
Wang et al.2016^56^	E:59	E:58.3 ± 10.9	E:37/22		√	NR	SQFZI 250 ml + XELOX	XELOX	14d × 3	③④⑤⑦
	C:59	C:59.4 ± 9.8	C:40/19							
Kong et al.2015^57^	E:39	E:52.4 ± 2.5	49/29		√	NR	CKSI 15 ml + XELOX	XELOX	5d × 8	⑦
	C:39	C:56.7 ± 3.5								
Liu.2020^58^	E:46	E:53.81 ± 4.01	E:24/22		√	NR	CKSI 15 ml + XELOX	XELOX	94d	①⑩
	C:46	C:54.09 ± 3.93	C:26/20							
Shi et al.2013^59^	E:46	E:56.76 ± 4.67	E:25/21	NR	NR	CI 50 ml + XELOX	XELOX	15d × 4	①②③④⑤⑧	
	C:40	C:55.74 ± 4.68	C:22/18							
Wu.2014^60^	E:29	E:69.37 ± 5.11	E:16/13		√	NR	KAI 60 ml + XELOX	XELOX	21d × 4	⑥⑦
	C:28	C:69.02 ± 5.35	C:15/13							
Wang.2015^61^	E:35	E:59.8 ± 16.3	E:20/15		√	NR	KAI 60 ml + XELOX	XELOX	14d × 2	①⑧⑩
	C:30	C:59.3 ± 14.7	C:20/10							
Gu et al.2017^62^	E:60	E:52.4 ± 3.4	E:37/23		√	E:(17.9 ± 3.6)m	XAPI 20-30 ml + XELOX	XELOX	14d × 4	①⑥⑦
	C:60	C:51.3 ± 3.6	C:35/25			C:(18.8 ± 3.4)m				
Du.2014^63^	E:49	E:60.27 ± 9.36	E:28/21		√	NR	KAI 60 ml + XELOX	XELOX	14d × 2	⑧⑨⑩
	C:46	C:56.4 ± 9.45	C:33/13							

Note: E, teatment group; C, control group; M,male; F, female; NR, not reported; SMYI, shengmaiyin injection; SMI, shenmai injection; KLT, kanglaite injection; SQFZI, shenqifuzheng injection; CI, cinobufacini injection; BJOI, brucea javanica oil injection; MI, matrine injection; XAPI, xiaoaiping injection; ADI, aidi injection; KAI, kangai injection; CKSI, Compound Kushen injection ① Clinical effectiveness rate; ② CD3^+^; ③ CD4^+^; ④ CD8^+^; ⑤ CD4+/CD8+; ⑥ KPS; ⑦ Gastrointestinal reactions; ⑧ Leukopenia; ⑨Platelet decline; ⑩ nausea and vomitting.

**FIGURE 3:**
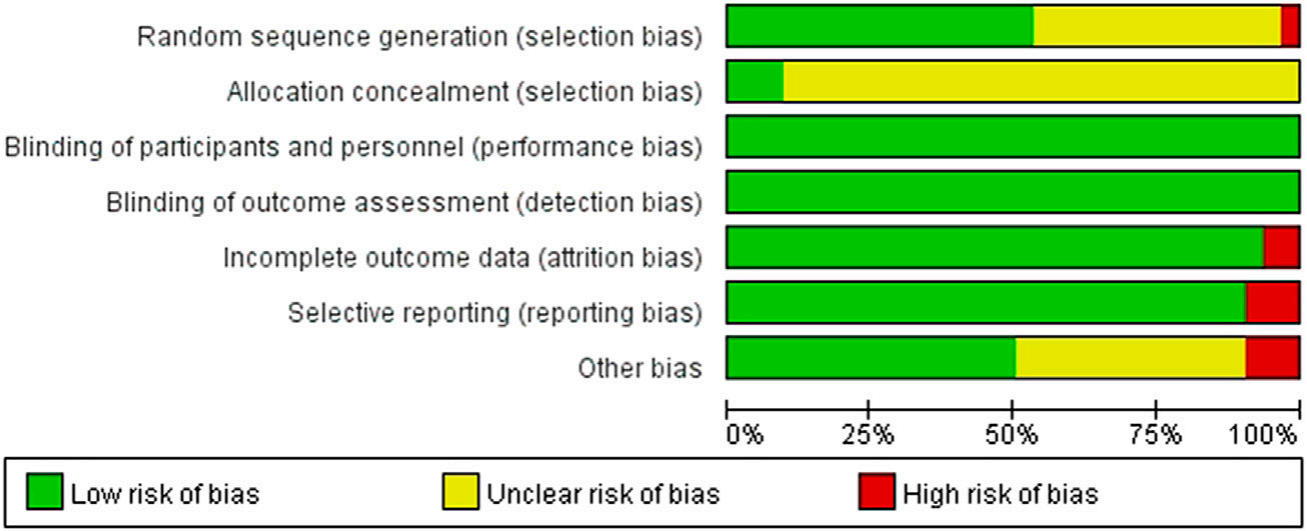
Risk of bias graph.

**FIGURE 4:**
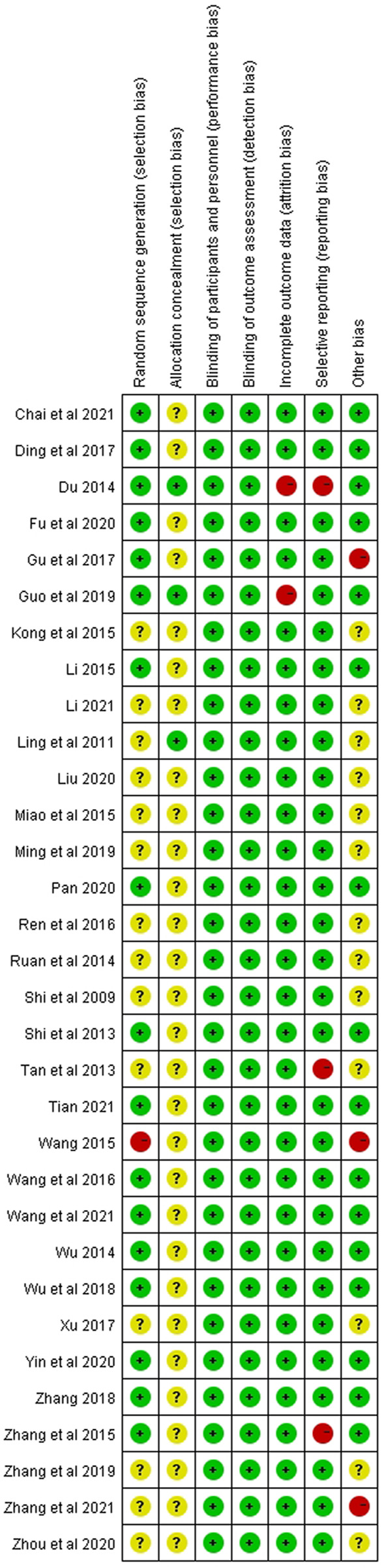
Risk of bias summary.

We critically appraised the methodological quality of the included RCTs in accordance with the Cochrane risk of bias tool. In random sequence generation, although all trials mentioned randomization, a total of 17 RCTs provided the details of the randomized grouping method; therefore, these trials were rated as low-risk. In particular, 14 RCTs used a random number table; 2 RCTs used block-randomized, multicenter, parallel-controlled designs; 1 RCT used the random number method; in addition, 1 RCT was classified as high-risk because the physicians grouped the patients according to their preferences. None of the RCTs referred to the method of blinding. Regarding allocation concealment, 2 RCTs used sealed opaque envelopes. There was also 1 RCT that used the paper bag method. There were 3 RCTs with incomplete outcomes in terms of selective reporting. In terms of incomplete outcome data, there were 2 RCTs with missing numbers in the control group greater than 10 percent. Among other biases, a total of 3 RCTs were rated as high-risk, 2 of which were statistically incorrect, and one RCT was rated as high-risk due to grouping by patient preference. In general, the methodological quality of the included RCTs was not high. A summary of the risk of bias for each included RCT is shown in Figure 3, 4.

### Outcomes

#### The clinical effectiveness rate

**FIGURE 5:**
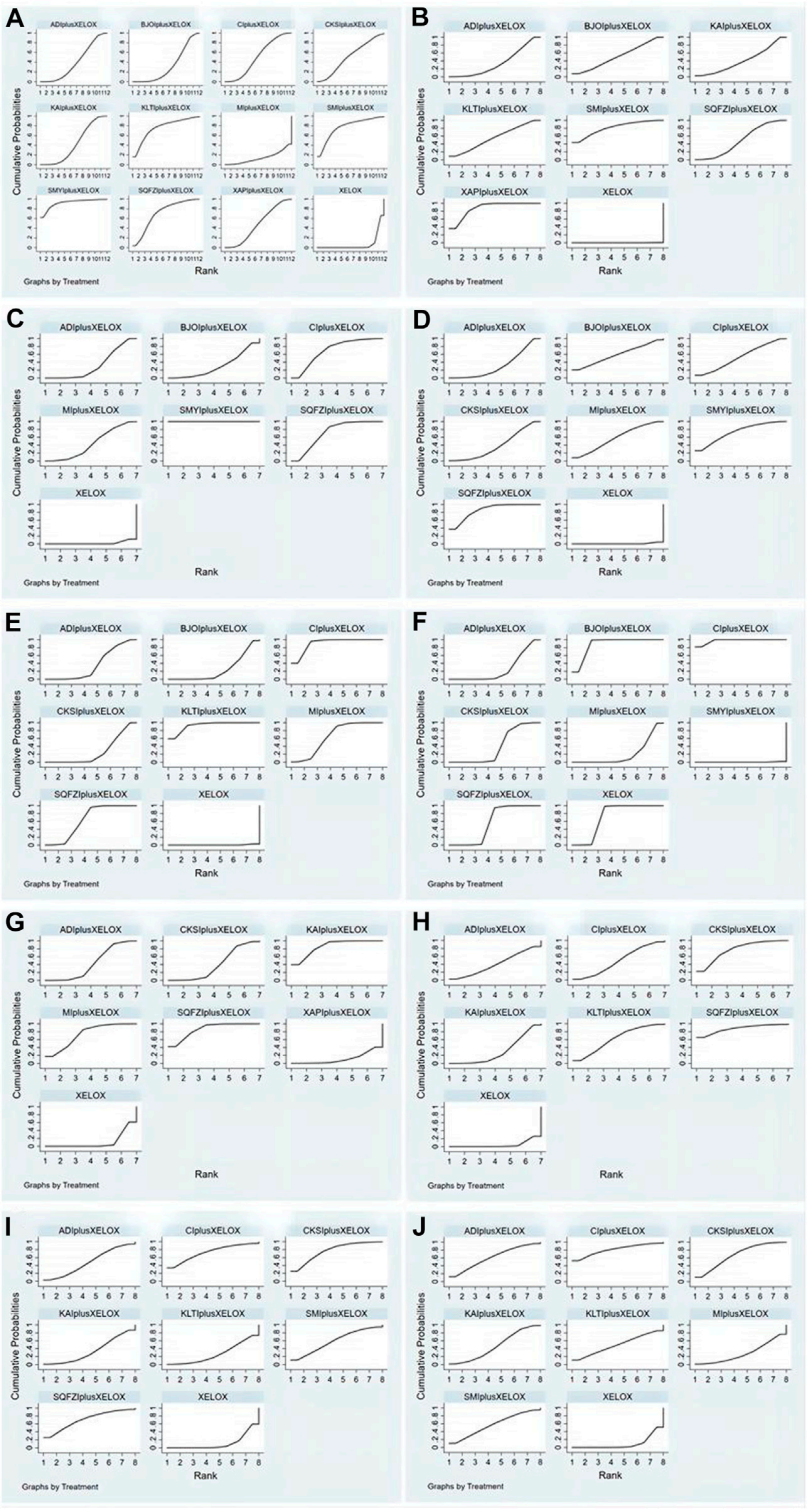
Rank of the cumulative probabilities for outcomes: **(A)** the clinical effectiveness rate; **(B)** performance status; **(C)** CD3^+^; **(D)** CD4^+^; **(E)** CD4+/CD8+; **(F)** CD8^+^; **(G)** Gastrointestinal reactions; **(H)** Leukopenia; **(I)** Platelet decline; **(J)** nausea and vomitting.

The data on the clinical effectiveness rate were available for 22 RCTs involving 11 types of CMIs. According to the results of the NMA illustrated in Table 2 (lower left section), RRs showed that, compared with the use of XELOX alone, combined with Shengmaiyin (RR = 2.42, 95%CIs: 1.25-5.35), Shenmai (RR = 1.77, 95%CIs: 1.03-3.25), Kanglaite (RR = 1.74,95%CIs:1.01-3.27),Shenqifuzheng(RR = 1.54, 95%CIs:1.09-2.24), Cinobufacini(RR = 1.29,95%CIs:1.07-1.6), Xiaoaiping (RR = 1.28,95%CIs:1.04-1.63), Kangai (RR = 1.26, 95%CIs: 1.1-1.48), Aidi (RR = 1.23, 95%CIs:1.03-1.49), Brucea Javanica Oil (RR = 1.18, 95%CIs: 1.01-1.43), on the basis of chemotherapy, could improve the clinical effectiveness rate and render the difference between groups statistically significant. In addition, there were statistical differences between the CMI groups; RRs showed that, compared with using Shengmaiyin + XELOX, Matrine (RR = 0.39, 95%CIs: 0.15-0.92) and Brucea Javanica Oil (RR = 0.49, 95%CIs: 0.22-0.97) performed poorly. After the ranking of each intervention’s efficacy, the combination of Shengmaiyin + XELOX (SUCRA91.8%) had the highest probability of providing the best treatment for CRC in terms of improving the clinical effectiveness rate, followed by the combination of Shenmai + XELOX (SUCRA76.8%) and the combination of Kanglaite + XELOX (SUCRA75.6%) (Figure 5A; Table 7 and Supplementary Figure S1A).

**TABLE 2:** Results of (MD/RR, 95% CI) Network Meta-Analysis for Improvement of Performance Status (Upper right section) and the Clinical Effective Rate (Lower left section).

ADI + XELOX	1.2 (-4.21, 6.62)	—	—	0.3 (-4.91, 5.48)	1.47 (-3.72, 6.66)	—	3.87 (-1.8, 9.53)	—	1.08 (-2.33, 4.5)	3.88 (0.8, 6.94)	-5.92 (-8.73, -3.12)
1.04 (0.8, 1.33)	BJOI + XELOX	—	—	-0.9 (-7.31, 5.46)	0.28 (-6.09, 6.62)	—	2.67 (-4.11, 9.44)	—	-0.12 (-5.11, 4.9)	2.68 (-2.14, 7.47)	**-7.11 (-11.75, -2.5)**
0.95 (0.72, 1.24)	0.91 (0.7, 1.19)	CI + XELOX	—	—	—	—	—	—	—	—	—
0.93 (0.63, 1.35)	0.9 (0.61, 1.3)	0.98 (0.66, 1.44)	CKSI + XELOX	—	—	—	—	—	—	—	—
0.97 (0.77, 1.23)	0.94 (0.75, 1.18)	1.02 (0.8, 1.32)	1.04 (0.73, 1.52)	KAI + XELOX	1.17 (-5, 7.36)	—	3.57 (-3.02, 10.18)	—	0.79 (-3.99, 5.54)	3.58 (-0.96, 8.14)	**-6.21 (-10.57, -1.84)**
0.71 (0.37, 1.26)	0.68 (0.36, 1.21)	0.74 (0.39, 1.33)	0.76 (0.37, 1.45)	0.73 (0.38, 1.28)	KLTI + XELOX	—	2.4 (-4.19, 9.01)	—	-0.39 (-5.18, 4.42)	2.41 (-2.19, 6.97)	**-7.39 (-11.79, -3.01)**
1.31 (0.74, 2.33)	1.26 (0.71, 2.24)	1.38 (0.77, 2.47)	1.4 (0.75, 2.67)	1.34 (0.77, 2.37)	1.86 (0.86, 4.23)	MI + XELOX	—	—	—	—	—
0.7 (0.37, 1.24)	0.67 (0.36, 1.19)	0.73 (0.39, 1.31)	0.75 (0.38, 1.43)	0.72 (0.38, 1.26)	0.99 (0.44, 2.25)	0.53 (0.24, 1.15)	SMI + XELOX	—	-2.79 (-8.12, 2.53)	0.01 (-5.11, 5.13)	**-9.8 (-14.74, -4.83)**
0.51 (0.23, 1.01)	**0.49 (0.22, 0.97)**	0.54 (0.24, 1.07)	0.55 (0.23, 1.16)	0.52 (0.23, 1.04)	0.72 (0.27, 1.79)	**0.39 (0.15, 0.92)**	0.73 (0.28, 1.81)	SMYI + XELOX	—	—	—
0.8 (0.53, 1.18)	0.77 (0.51, 1.13)	0.84 (0.55, 1.26)	0.86 (0.52, 1.4)	0.82 (0.55, 1.2)	1.13 (0.58, 2.31)	0.61 (0.32, 1.16)	1.14 (0.59, 2.31)	1.57 (0.73, 3.7)	SQFZI + XELOX	**2.8 (0.47, 5.11)**	**-7 (-8.95, -5.06)**
0.96 (0.71, 1.27)	0.92 (0.69, 1.23)	1.01 (0.75, 1.36)	1.03 (0.69, 1.54)	0.99 (0.75, 1.28)	1.36 (0.75, 2.64)	0.73 (0.41, 1.31)	1.37 (0.76, 2.63)	1.88 (0.93, 4.29)	1.2 (0.79, 1.85)	XAPI + XELOX	**-9.8 (-11.06, -8.52)**
**1.23 (1.03, 1.49)**	**1.18 (1.01, 1.43)**	**1.29 (1.07, 1.6)**	1.32 (0.96, 1.87)	**1.26 (1.1, 1.48)**	**1.74 (1.01, 3.27)**	0.94 (0.55, 1.61)	**1.77 (1.03, 3.25)**	**2.42 (1.25, 5.35)**	**1.54 (1.09, 2.24)**	**1.28 (1.04, 1.63)**	XELOX

The values in bold are values with statistical analysis significance.

#### Performance status

In total, 8 RCTs with 7 CMIs contributed to the analysis of performance status.According to the results of theNMA illustrated in Table 2 (upper right section), taking Aidi + XELOX, Brucea Javanica Oil + XELOX, Kangai + XELOX, Kanglaite + XELOX, Shenmai + XELOX, Shenqifuzheng + XELOX, and Xiaoaiping + XELOX as the control group, the efficacy of XELOX alone is inferior to the above groups. These results were statistically significant; the MD values and 95% CIs were -5.92(-8.73,-3.12), -7.11 (-11.75, -2.5), -6.21 (-10.57, -1.84), -7.39 (-11.79, -3.01),-9.8 (-14.74,-4.83), -7 (-8.95, -5.06), and -9.8 (-11.06, -8.52), respectively. In addition, MD values showed that, compared with the use of Aidi + XELOX, Xiaoaiping (MD = 3.88,95%CIs:0.8-6.94) could improve the performancestatus; MD values showed that, compared with the use of Shenqifuzheng + XELOX, Xiaoaiping (MD = 2.8,95%CIs:0.47-5.11) could improve the performance status. After the ranking of each intervention’s efficacy, the combination of Xiaoaiping + XELOX (SUCRA87.6%) had the highest probability of providing the best treatment for CRC in terms of improving the performance status, followed by the combination of Shenmai + XELOX (SUCRA80.8%) and the combination of Kanglaite + XELOX (SUCRA55.3%) (Figure 5B; Table 7 and Supplementary Figure S1B).

##### CD4^+^

In total, 12 RCTs with 7 CMIs contributed to the analysis of CD4. According to the results of the NMA illustrated in Table 3 (lower Left section), MD values showed, that compared with the use of XELOX alone, combination with Shenqifuzheng (MD = 9.08,95%CIs:7.84-10.31), Shengmaiyin (MD = 8.31,95%CIs:5.64-10.97), BruceaJavanica Oil (MD = 7.03,95%CIs:1-13.06), Matrine (MD = 6.79,95%CIs:4.79-8.79), Cinobufacini (MD = 6.3,95%CIs:4.03-8.57), Compound Kushen (MD = 5.19,95%CIs:3.83-6.54), and Aidi (MD = 3.79,95%CIs:2.96-4.62), on the basis of chemotherapy, could increase the value of CD4^+^ and render the difference betweengroups statistically significant. Moreover, there were statistical differences between the CMI groups, and MD values showed that, compared with the use of Shenqifuzheng + XELOX, Compound Kushen (MD = -3,89,95%CIs: 5.71∼-2.04), Cinobufacini (MD = -2.78,95%CIs: 5.36∼-0.18), and Aidi (MD = -5.29,95%CIs: 6.78∼-3.79) performed poorly; MDs showed that compared with the use of Shengmaiyin + XELOX, Compound Kushen (MD = -3.12,95%CIs: 6.11∼-0.13) and Aidi (MD = -4.52,95%CIs: 7.31∼-1.72) performed poorly; MD values showed that compared to the use of Matrine + XELOX, Aidi (MD = -3,95%CIs: 5.17∼-0.84) performed poorly; MD values showed that compared to the use of Cinobufacini + XELOX, Aidi (MD = -2.51,95%CIs: 4.93∼-0.08) performed poorly. After the ranking of each intervention’s efficacy, the combination of Shenqifuzheng + XELOX (SUCRA85.5%) had the highest probability of providing the best treatment for CRC in terms of increasing the value of CD4^+^, followed by the combination of Shengmaiyin + XELOX (SUCRA73.1%) and the combination of BruceaJavanica Oil + XELOX (SUCRA58.8%) (Figure 5D; Table 7 and Supplementary Figure S1D).

**TABLE 3:** Results (MD, 95% CI) of Network Meta-Analysis for CD8^+^ (Upper Rightsection) and CD4^+^ (Lower Left section).

ADI + XELOX	9.09 (6, 12.21)	10.99 (8.16, 13.86)	1.24 (-0.58, 3.06)	-0.5 (-2.9, 1.9)	-3.8 (-5.7, -1.91)	3.04 (1.02, 5.04)	5.4 (3.79, 7)
-3.24 (-9.32, 2.84)	BJOI + XELOX	1.9 (-1.66, 5.46)	**-7.85 (-10.66, -5.06)**	**-9.6 (-12.81, -6.4)**	**-12.9 (-15.75, -10.05)**	**-6.06 (-8.99, -3.14)**	**-3.7 (-6.37, -1.04)**
**-2.51 (-4.93, -0.08)**	0.73 (-5.71, 7.18)	CI + XELOX	**-9.75 (-12.26, -7.25)**	**-11.49 (-14.47, -8.55)**	**-14.8 (-17.37, -12.24)**	**-7.96 (-10.59, -5.31)**	**-5.6 (-7.96, -3.25)**
-1.4 (-2.99, 0.18)	1.85 (-4.33, 8.01)	1.11 (-1.55, 3.75)	CKSI + XELOX	-1.74 (-3.73, 0.25)	**-5.05 (-6.36, -3.71)**	**1.8 (0.32, 3.29)**	**4.16 (3.28, 5.03)**
**-3 (-5.17, -0.84)**	0.25 (-6.12, 6.58)	-0.5 (-3.52, 2.54)	-1.61 (-4.02, 0.81)	MI + XELOX	**-3.3 (-5.34, -1.25)**	**3.54 (1.39, 5.7)**	**5.9 (4.11, 7.68)**
**-4.52 (-7.31, -1.72)**	-1.26 (-7.88, 5.3)	-2.01 (-5.5, 1.49)	**-3.12 (-6.11, -0.13)**	-1.51 (-4.84, 1.83)	SMYI + XELOX	**6.84 (5.28, 8.41)**	**9.2 (8.19, 10.2)**
**-5.29 (-6.78, -3.79)**	-2.04 (-8.18, 4.1)	**-2.78 (-5.36, -0.18)**	**-3.89 (-5.71, -2.04)**	-2.28 (-4.63, 0.07)	-0.77 (-3.71, 2.17)	SQFZI + XELOX	**2.36 (1.15, 3.56)**
**3.79 (2.96, 4.62)**	**7.03 (1, 13.06)**	**6.3 (4.03, 8.57)**	**5.19 (3.83, 6.54)**	**6.79 (4.79, 8.79)**	**8.31 (5.64, 10.97)**	**9.08 (7.84, 10.31)**	XELOX

The values in bold are values with statistical analysis significance.

##### CD8^+^

There were 10 RCTs with 7 CMIs that contributed to the analysis of CD8^+^. According to the results of the NMA illustrated in Table 3 (upper right section), MD values showed that compared with the use of Aidi + XELOX, Brucea Javanica Oil (MD = 9.09,95%CIs:6-12.21), Cinobufacini (MD = 10.99,95%CIs:8.16-13.86), Shenqifuzheng (MD = 3.04,95%CIs:1.02-5.04), and XELOX (MD = 5.4,95%CIs:3.79-7) could improve the value of CD8^+^, but the increase in Shengmaiyin (MD = -3.8,95%CIs: 5.7∼-1.91) was lower than that of Aidi + XELOX. MD values showedthat compared with using Brucea JavanicaOil + XELOX, Compound Kushen (MD = -7.85,95%CIs: 10.66∼-5.06), Matrine (MD = -9.6,95%CIs: 12.81∼-6.4), Shengmaiyin (MD = -12.9,95%CIs: 15.75∼-10.05), Shenqifuzheng (MD = -6.06,95%CIs: 8.99∼-3.14), and XELOX (MD = -3.7,95%CIs: 6.37∼-1.04) were worse; MD values showed that compared with using Cinobufacini + XELOX, Compound Kushen (MD = -9.75,95%CIs: 12.26∼-7.25), Matrine (MD = -11.49,95%CIs: 14.47∼-8.55), Shengmaiyin (MD = -14.8,95%CIs: 17.37∼-12.24), Shenqifuzheng (MD = -7.96,95%CIs: 10.59∼-5.31), and XELOX (MD = -5.6,95%CIs: 7.96∼-3.25) were worse. MD values showed that compared with using Compound Kushen, Shenqifuzheng (MD = 1.8,95%CIs:0.32-3.29) and XELOX (MD = 4.16,95%CIs:3.28-5.03) could improve the value of CD8^+^, but the increase in Shengmaiyin (MD = -5.05,95%CIs: 6.36∼-3.71) was worse than Compound Kushen + XELOX. MD values showed that compared with using Matrine + XELOX, Shenqifuzheng (MD = 3.54,95%CIs:1.39-5.7) and XELOX (MD = 5.9,95%CIs:4.11-7.68) could improve the value of CD8^+^, but the increase in Shengmaiyin (MD = -3.3,95%CIs: 5.34∼-1.25) was worse than Matrine + XELOX. MD values showed that compared with using Shengmaiyin + XELOX, Shenqifuzheng (MD = 6.84,95%CIs:5.28-8.41) and XELOX (MD = 9.2,95%CIs:8.19-10.2) could improve the value of CD8^+^. MD values showed that compared with using Shenqifuzheng + XELOX, XELOX(MD = 2.36,95%CIs:1.15-3.56) could improve the value of CD8^+^. After the ranking of each intervention’s efficacy, the combination of Cinobufacini + XELOX (SUCRA97.4%) had the highest probability of providing the best treatment for CRC in terms of increasing the value of CD8^+^, followed by the combination of Brucea Javanica Oil + XELOX (SUCRA88.2%) and XELOX (SUCRA71.3%) (Figure 5F; Table 7 and Supplementary Figure S1F).

##### CD3^+^

A total of 7 RCTs with 6 CMIs contributed to the analysis of CD3^+^. According to the results of the NMA illustrated in Table 4 (upper right section), MD values showed that, compared with using Aidi + XELOX, Cinobufacini(MD = 6.18,95%CIs:0.86-11.49), Shenqifuzheng (MD = 6.01,95%CIs:4.2-7.82), and Shengmaiyin (MD = 19.44,95%CIs:17.29-21.58) could improve the value of CD3^+^, but the increase in XELOX (MD = -4.54,95%CIs: 5.74∼-3.34) was worse than Aidi + XELOX. MD values showed that compared with using Brucea Javanica Oil + XELOX, Shenqifuzheng (MD = 6.21,95%CIs:0.48-11.89) and Shengmaiyin (MD = 19.65,95%CIs:13.78-25.43) could improve the value of CD3^+^. MD values showed that compared with using Cinobufacini + XELOX, Shengmaiyin (MD = 13.26,95%CIs:7.78-18.76) could improve the value of CD3^+^, but the increase in XELOX (MD = -10.72,95%CIs: 15.9∼-5.53) was worse than Cinobufacini + XELOX. MD values showed that compared with using Matrine + XELOX, Shenqifuzheng (MD = 4.06,95%CIs:1.36-6.75) and Shengmaiyin (MD = 17.49,95%CIs:14.55-20.41) could improve the value of CD3^+^, but the increase in XELOX (MD = -6.49,95%CIs: 8.81∼-4.17) was worse than Matrine + XELOX. MD values showed that compared with using Shenqifuzheng + XELOX, Shengmaiyin (MD = 13.43,95%CIs:11.19-15.66) could improve the value of CD3^+^, but the increase in XELOX (MD = -10.55,95%CIs: 11.91∼-9.19) was worse.MD values showed that, compared with using Shengmaiyin + XELOX, XELOX (MD = -23.98,95%CIs: 25.75∼-22.2) performed poorly. After the ranking of each intervention’s efficacy, the combination of Shengmaiyin + XELOX (SUCRA100.0%) had the highest probability of providing the best treatment for CRC in terms of increasing the value of CD3^+^, followed by the combination of Shenqifuzheng + XELOX (SUCRA71.3%) and Cinobufacini (SUCRA69.7%) (Figure 5C; Table 7 and Supplementary Figure S1C).

**TABLE 4:** Results (RR, 95% CI) of Network Meta-Analysis for CD3^+^ (Upper Right section) and CD4+/CD8+ (Lower Left section).

ADI + XELOX	-0.2 (-5.85, 5.49)	6.18 (0.86,11.49)	—	—	1.95 (-0.66,4.58)	6.01 (4.2, 7.82)	19.44(17.29,21.5)	-4.54 (-5.74, -3.34)
0.09 (-0.06, 0.24)	BJOI + XELOX	6.38(-1.25,13.94)	—	—	2.16 (-3.88,8.17)	**6.21 (0.48,11.89)**	**19.65(13.78,25.4)**	-4.34 (-9.91, 1.19)
**-0.48 (-0.64,-0.32)**	**-0.57 (-0.75,-0.39)**	CI + XELOX	—	—	-4.23(-9.92,1.45)	-0.17(-5.53,5.19)	**13.26 (7.78,18.76)**	**-10.72 (-15.9, -5.53)**
0.09 (-0.03, 0.22)	0 (-0.14, 0.14)	**0.57 (0.42, 0.73)**	CKSI + XELOX	—	—	—	—	—
**-0.53 (-0.83,-0.23)**	**-0.62 (-0.93,-0.31)**	-0.05 (-0.37,0.27)	**-0.62 (-0.92,-0.33)**	KLTI + XELOX	—	—	—	—
**-0.21 (-0.35,-0.07)**	**-0.3 (-0.46, -0.14)**	**0.27 (0.1, 0.44)**	**-0.3 (-0.44, -0.17)**	**0.32 (0.01, 0.63)**	MI + XELOX	**4.06 (1.36, 6.75)**	**17.49(14.55,20.4)**	**-6.49 (-8.81, -4.17)**
**-0.24 (-0.34,-0.13)**	**-0.33 (-0.45, -0.2)**	**0.24 (0.1, 0.39)**	**-0.33 (-0.43,-0.24)**	0.29 (0, 0.58)	-0.03(-0.15,0.09)	SQFZI + XELOX	**13.43(11.19,15.6)**	**-10.55(-11.91,-9.19)**
—	—	—	—	—	—	—	SMYI + XELOX	**-23.98(-25.75,-22.2)**
**0.3 (0.21, 0.39)**	**0.21 (0.1, 0.32)**	**0.78 (0.64, 0.91)**	**0.21 (0.12, 0.29)**	**0.83 (0.54, 1.12)**	**0.51 (0.4, 0.62)**	**0.54 (0.49, 0.59)**	—	XELOX

The values in bold are values with statistical analysis significance.

##### CD4+/CD8+

A total of 12 RCTs with 7 CMIs contributed to the analysis of CD4+/CD8+. According to the results of the NMA illustrated in Table 4 (lower Left section), MD values showed that, compared to using XELOX alone, combination with Kanglaite (MD = 0.83,95%CIs:0.54-1.12), Cinobufacini (MD = 0.78,95%CIs:0.64-0.91), Matrine (MD = 0.51,95%CIs:0.4-0.62), Shenqifuzheng (MD = 0.54,95%CIs:0.49-0.59), Aidi (MD = 0.3,95%CIs:0.21-0.39), Brucea Javanica Oil (MD = 0.21,95%CIs:0.1-0.32), and Compound Kushen (MD = 0.21,95%CIs:0.12-0.29) on the basis of chemotherapy could increase the rate of CD4+/CD8+ and render the difference betweengroups statistically significant. Moreover, there were statistical differences between the CMI groups, and MD values showed that, compared to using Shenqifuzheng + XELOX, Cinobufacini (MD = 0.24,95%CIs:0.1-0.39) could increase the rate of CD4+/CD8+, but the increases in Compound Kushen (MD = -0.33,95%CIs: 0.43∼-0.24), Brucea Javanica Oil (MD = -0.33,95%CIs: 0.45∼-0.2), and Aidi (MD = -0.24,95%CIs: 0.34∼-0.13) were worse than Shenqifuzheng + XELOX. MD values showed that compared to using Matrine + XELOX, Kanglaite (MD = 0.32,95%CIs:0.01–0.63)and Cinobufacini(MD = 0.27,95%CIs:0.1-0.44) could increase the rate of CD4+/CD8+, but the increases in Compound Kushen (MD = -0.3,95%CIs: 0.44∼-0.17), Brucea Javanica Oil (MD = -0.3,95%CIs: 0.46∼-0.14), and Aidi (MD = -0.21,95%CIs: 0.35∼-0.07) were worse than Matrine + XELOX; MD values showed that compared tousing Kanglaite + XELOX, Compound Kushen (MD = -0.62,95%CIs: 0.92∼-0.33), Brucea Javanica Oil (MD = -0.62,95%CIs: 0.93∼-0.31), and Aidi (MD = -0.53,95%CIs: 0.83∼-0.23) were worse than Kanglaite + XELOX; MD values showed that compared tousing Compound Kushen + XELOX, Cinobufacini (MD = 0.57,95%CIs:0.42-0.73) could increase the rate of CD4+/CD8+; MD values showed that compared to using Cinobufacini + XELOX, Brucea Javanica Oil (MD = -0.57,95%CIs: 0.75∼-0.39) and Aidi (MD = -0.48,95%CIs: 0.64∼-0.32) were worse. After the ranking of each intervention’s efficacy, the combination of Kanglaite + XELOX (SUCRA92.8%) had the highest probability of providing the best treatment for CRC in terms of increasing the rate of CD4+/CD8+, followed by the combination of Cinobufacini + XELOX (SUCRA90.8%) and the combination of Matrine + XELOX (SUCRA64.6%) (Figure 5E; Table 7 and Supplementary Figure S1E).

#### Gastrointestinal reactions

A total of 10 RCTs with 6 CMIs contributed to the analysis of Gastrointestinal reactions. According to the results of the NMA illustrated in Table 5 (upper right section), RR values showed that, compared with using Aidi + XELOX, Kangai(RR = 0.53,95%CIs:0.3-0.91) and Shenqifuzheng (RR = 0.53,95%CIs:0.28-0.95) could effectively relieve Gastrointestinal reactions, but the incidence of XELOX (RR = 1.48,95%CIs:1.07-2.13) was higher than Aidi + XELOX.RR values showed that compared with using Compound Kushen + XELOX, Kangai (RR = 0.51,95%CIs:0.28-0.91) and Shenqifuzheng (RR = 0.51,95%CIs:0.27-0.95) could effectively relieve Gastrointestinal reactions. RR values showed that compared with using Kangai + XELOX, Xiaoaiping (RR = 3.08,95%CIs:1.35-7.22) and XELOX(RR = 2.82,95%CIs:1.87-4.48) were worse. RR values showed that compared with using Matrine + XELOX, Xiaoaiping(RR = 2.53,95%CIs:1.03-6.53) and XELOX(RR = 2.31,95%CIs:1.37-4.35) were worse.RR values showed that compared with using Shenqifuzheng + XELOX, Xiaoaiping (RR = 3.07,95%CIs:1.3-7.44) and XELOX(RR = 2.8,95%CIs:1.76-4.75) were worse. After the ranking of each intervention’s efficacy, the combination of Shenqifuzheng + XELOX (SUCRA86.1%) had the highest probability of providing the best treatment for CRC in terms of improving Gastrointestinal reactions, followed by the combination of Kangai + XELOX (SUCRA85.7%) and Matrine + XELOX (SUCRA64.6%) (Figure 5G; Table 7 and Supplementary Figure S1G).

**TABLE 5:** Results (MD, 95% CI) of Network Meta-Analysis for Gastrointestinal reactions (Upper Right section) and Leukopenia (Lower Left section).

ADIplusXELOX	—	1.04 (0.61, 1.75)	0.53 (0.3, 0.91)	—	0.64 (0.32, 1.21)	0.53 (0.28, 0.95)	1.62 (0.74, 3.62)	1.48 (1.07, 2.13)
1.12 (0.34, 3.38)	CIplusXELOX	—	—	**—**	—	—	—	—
1.98 (0.54, 7.31)	1.77 (0.64, 5.29)	CKSIplusXELOX	**0.51 (0.28, 0.91)**	**—**	0.62 (0.3, 1.21)	**0.51 (0.27, 0.95)**	1.56 (0.69, 3.57)	1.42 (0.98, 2.17)
0.87 (0.3, 2.26)	0.78 (0.4, 1.46)	0.44 (0.17, 1.01)	KAIplusXELOX	**—**	1.22 (0.58, 2.45)	1 (0.51, 1.94)	**3.08 (1.35, 7.22)**	**2.82 (1.87, 4.48)**
1.41 (0.41, 4.56)	1.26 (0.51, 3.27)	0.71 (0.23, 2.12)	1.61 (0.8, 3.6)	KLTIplusXELOX	—	—	—	—
—	—	—	—	—	MIplusXELOX	0.82 (0.39, 1.81)	**2.53 (1.03, 6.53)**	**2.31 (1.37, 4.35)**
3.53 (0.63, 30.84)	3.12 (0.71, 25.27)	1.77 (0.35, 14.89)	**3.99 (1.03, 30.37)**	2.48 (0.54, 20.43)	—	SQFZIplusXELOX	**3.07 (1.3, 7.44)**	**2.8 (1.76, 4.75)**
—	—	—	—	—	—	—	XAPIplusXELOX	0.92 (0.45, 1.87)
0.61 (0.21, 1.52)	**0.55 (0.29, 0.97)**	**0.31 (0.12, 0.68)**	**0.7 (0.54, 0.9)**	**0.44 (0.2, 0.83)**	—	**0.18 (0.02, 0.66)**	—	XELOX

The values in bold are values with statistical analysis significance.

#### Leukopenia

A total of 9 RCTs with 6 CMIs contributed to the analysis of Leukopenia. According to the results of the NMA illustrated in Table 5 (lower Left section), RRs showed that, compared with the use of XELOX alone, combined with Cinobufacini (RR = 0.55, 95%CIs: 0.29-0.97), Compound Kushen (RR = 0.31, 95%CIs: 0.12-0.68), Kangai (RR = 0.7, 95%CIs:0.54-0.9), Kanglaite (RR = 0.44, 95%CIs:0.2-0.83), Shenqifuzheng (RR = 0.18,95%CIs:0.02-0.66), on the basis of chemotherapy, could effectively relieve the Leukopenia and render the difference between groups statistically significant. In addition, there were statistical differences between the CMI groups; RRs showed that, compared with using Shenqifuzheng + XELOX, Kangai (RR = 3.99, 95%CIs: 1.03-30.37) performed poorly. After the ranking of each intervention’s efficacy, the combination of Shenqifuzheng + XELOX (SUCRA87.5%) had the highest probability of providing the best treatment for CRC in terms of improving the Leukopenia, followed by the combination of Compound Kushen + XELOX (SUCRA76.6%) and the combination of Kanglaite + XELOX (SUCRA61.5%) (Figure 5H; Table 7 and Supplementary Figure S1H).

#### Platelet decline

A total of 9 RCTs with 7 CMIs contributed to the analysis of Platelet decline. According to the results of the NMA illustrated in Table 6 (upper right section), taking Aidi + XELOX, Cinobufacini + XELOX, Compound Kushen + XELOX, Shenmai + XELOX, and Shenqifuzheng + XELOX as the control group, the incidence of Platelet decline were higher with XELOX alone than the above groups. These results were statistically significant; the RR values and 95% CIs were 2.13(1.16,4.25), 4.49 (1.15, 30.65), 4.09 (2.13, 8.78), 2.7 (1.12, 7.93), 3.97 (1.31,18.27), respectively. In addition, RR values showed that, compared with the use of Compound Kushen + XELOX, Kangai (RR = 2.61,95%CIs:1.09-6.59) and Kanglaite (RR = 3.22,95%CIs:1.41-7.92) performed poorly. After the ranking of each intervention’s efficacy, the combination of Compound Kushen + XELOX (SUCRA76.7%) had the highest probability of providing the best treatment for CRC in terms of improving Platelet decline, followed by the combination of Cinobufacini + XELOX (SUCRA73.1%) and Shenqifuzheng + XELOX (SUCRA70.5%) (Figure 5I; Table 7 and Supplementary Figure S1I).

**TABLE 6:** Results (MD, 95% CI) of Network Meta-Analysis for Platelet decline (Upper Right section) and nausea and vomitting (Lower Left section).

ADIplusXELOX	0.47 (0.06, 2.22)	0.52 (0.2, 1.35)	1.35 (0.58, 3.25)	1.68 (0.76, 3.86)	—	0.79 (0.23, 2.41)	0.54 (0.1, 1.99)	2.13 (1.16, 4.25)
1.78 (0.45, 8.91)	CIplusXELOX	1.1 (0.23, 8.19)	2.87 (0.65, 20.68)	3.55 (0.82, 24.96)	—	1.67 (0.29, 13.31)	1.13 (0.15, 10.28)	**4.49 (1.15, 30.65)**
1.26 (0.5, 2.93)	0.71 (0.15, 2.34)	CKSIplusXELOX	**2.61 (1.09, 6.59)**	**3.22 (1.41, 7.92)**	—	1.52 (0.43, 4.85)	1.03 (0.2, 3.99)	**4.09 (2.13, 8.78)**
0.7 (0.29, 1.55)	0.4 (0.09, 1.25)	0.56 (0.33, 0.93)	KAIplusXELOX	1.24 (0.59, 2.62)	—	0.58 (0.18, 1.67)	0.39 (0.08, 1.39)	1.57 (0.91, 2.8)
0.83 (0.23, 3.12)	0.46 (0.08, 2.23)	0.65 (0.23, 2.16)	1.17 (0.42, 3.74)	KLTIplusXELOX	—	0.47 (0.15, 1.31)	0.32 (0.06, 1.09)	1.27 (0.79, 2.13)
0.57 (0.24, 1.23)	0.32 (0.07, 1.00)	0.45 (0.28, 0.73)	0.81 (0.55, 1.21)	0.69 (0.22, 1.87)	MIplusXELOX	—	—	—
0.95 (0.36, 2.37)	0.53 (0.11, 1.86)	0.75 (0.38, 1.52)	1.34 (0.74, 2.61)	1.15 (0.33, 3.51)	1.65 (0.93, 3.1)	SMIplusXELOX	0.68 (0.12, 3.17)	**2.7 (1.12, 7.93)**
—	—	—	—	—	—	—	SQFZIplusXELOX	**3.97 (1.31, 18.27)**
0.45 (0.19, 0.91)	**0.25 (0.06, 0.75)**	**0.35 (0.23, 0.53)**	**0.63 (0.46, 0.86)**	0.54 (0.18, 1.41)	**0.78 (0.6, 0.97)**	**0.47 (0.26, 0.78)**	—	XELOX

The values in bold are values with statistical analysis significance.

**TABLE 7:** SUCRA values of different groups for outcomes.

	The clinical effectiveness rate (%)	Performance status	CD3^+^	CD4^+^	CD8^+^	CD4^+^/CD8^+^	Gastrointestinal reactions	Leukopenia	Platelet decline	Nausea and vomitting
ADI + XELOX	39.9	35.0%	32.8%	31.7%	25.6%	36.7%	43.1%	40.6%	48.5%	**61.1%**
SQFZI + XELOX	70.3	48.8%	**71.3%**	**85.5%**	56.6%	63.6%	**86.1%**	**87.5%**	**70.5%**	—
SMI + XELOX	**76.8**	**80.8%**	—	—	—	—	—	—	58.9%	57.2%
KAI + XELOX	42.5	41.1%	—	—	—	—	**85.7%**	30.5%	34.1%	46.4%
KLTI + XELOX	**75.6**	**55.3%**	—	—	—	**92.8%**	—	**61.5%**	26.7%	49.6%
MI + XELOX	14.1	—	43.9%	58.0%	20.9%	**64.6%**	**73.5%**	—	—	28.4%
SMYI + XELOX	**91.8**	—	**100.0%**	**73.1%**	0.2%	—	—	—	—	—
CI + XELOX	50.4	—	**69.7%**	53.3%	**97.4%**	**90.8%**	—	48.8%	**73.1%**	**80.8%**
BJOI + XELOX	32.5	51.4%	30.3%	**58.8%**	**88.2%**	24.6%	—	—	—	—
CKSI + XELOX	50.0	—	—	38.9%	39.8%	26.4%	39.5%	**76.6%**	**76.7%**	**67.2%**
XAPI + XELOX	48.0	**87.6%**	—	—	—	—	11.4%	—	—	—
XELOX	7.0	0.1%	2.0%	0.7%	**71.3%**	0.4%	10.7%	4.6%	11.5%	9.2%

The values in bold are values with statistical analysis significance.

#### Nausea and vomitting

A total of 13 RCTs with 7 CMIs contributed to the analysis of nausea and vomitting. According to the results of the NMA illustrated in Table 6 (lower Left section), RRs showed that, compared with the use of XELOX alone, combined with Cinobufacini (RR = 0.25, 95%CIs: 0.06-0.75), Compound Kushen (RR = 0.35, 95%CIs: 0.23-0.53), Kangai (RR = 0.63, 95%CIs:0.46-0.86), Matrine (RR = 0.78, 95%CIs:0.6-0.97), Shenmai (RR = 0.47,95%CIs:0.26-0.78), on the basis of chemotherapy, could reduce the incidence of nausea and vomitting and render the difference between groups statistically significant. After the ranking of each in tervention’s efficacy, the combination of Cinobufacini + XELOX (SUCRA80.8%) had the highest probability of providing the best treatment for CRC in terms of improving nausea and vomitting, followed by the combination of Compound Kushen + XELOX (SUCRA67.2%) and Aidi + XELOX (SUCRA61.1%) (Figure 5J; Table 7 and Supplementary Figure S1J).

#### Adverse reactions

There are many adverse reactions to cancer, in the case of colorectal cancer, the main adverse reactions are gastrointestinal reactions, nausea and vomiting, Leukopenia and Platelet decline (Li et al., 2022). A total of 23 RCTs documented adverse reactions in this paper, in addition to the above adverse reactions, there are granulocytopenia, abdominal pain and diarrhea, hand–foot syndrome, neurotoxicity, Myelosuppression, and so on. Overall, the incidence of adverse reactions of Chinese medicine injections combined with XELOX was low. The results are shown in Table 8.

**TABLE 8:** Summary of results of adverse events for all interventions.The numberis the number of people with various adverse reactions. Case (%).

Intervention	ADI + XELOX	CKSI + XELOX	SQFZI + XELOX	SMI + XELOX	KAI + XELOX	KLTI + XELOX	CI + XELOX	XAPI + XELOX	MI + XELOX
Sample size	113	274	125	40	288	33	81	60	40
Gastrointestinal reactions	25(22.12%)	19(6.93%)	17(13.60%)	—	20(6.94%)	—	—	13(21.67%)	11(27.50%)
Granulocytopenia	23(20.35%)	—	—	15(37.50%)	23(7.99%)	—	—	—	—
Platelet decline	11(9.73%)	9(3.28%)	3(2.40%)	5(12.50)	16(5.56%)	15(45.45%)	2(2.47%)	—	38(95.00%)
Abdominal pain and diarrhea	5(4.42%)	12(4.38%)	—	—	5(1.74%)	—	2(2.47%)	—	—
Leukopenia	5(4.42%)	7(2.55%)	2(1.60%)	—	47(16.32%)	8(24.24%)	12(14.81%)	—	37(92.50%)
Alopecia	3(2.65%)	20(7.30%)	—	—	—	—	—	—	27(67.50%)
nausea and vomitting	4(3.54%)	25(9.12%)	—	12(30.00)	43(14.93%)	5(15.15%)	3(3.70%)		28(70.00%)
Liver and kidney abnormalities	—	—	7(5.60%)	—	21(7.29%)	14(42.42%)	1(1.23%)	6(10.00%)	24(60.00%)
Neurotoxicity	—	1(0.36%)	—	—	5(1.74%)	11(33.33%)	—	—	—
Myelosuppression	—	23(8.39%)	12(9.60%)	—	13(4.51%)	—	—	8(13.33%)	—
Hemoglobin reduction	—	—	—	—	13(4.51%)	7(21.21%)	—	—	—
Peripheral neuropathy	9(7.96%)	—	—	—	3(1.04%)	—	—	—	—
Hand-foot syndrome	3(2.65%)	—	—	—	33(11.46%)	8(24.24%)	1(1.23%)		
Other	Dizziness(5), Constipation(3)	Decreased quality of life(24), Dizziness(2),weakness(22)	—	—	Chest tightness and shortness of breath(1), Skin rash(2), Blood System(17),Anaphylaxis(1)	Electrocardiographic changes(1)	—	No details(5)	Erythrocyte reduction(32)

#### Cluster analysis

**FIGURE 6:**
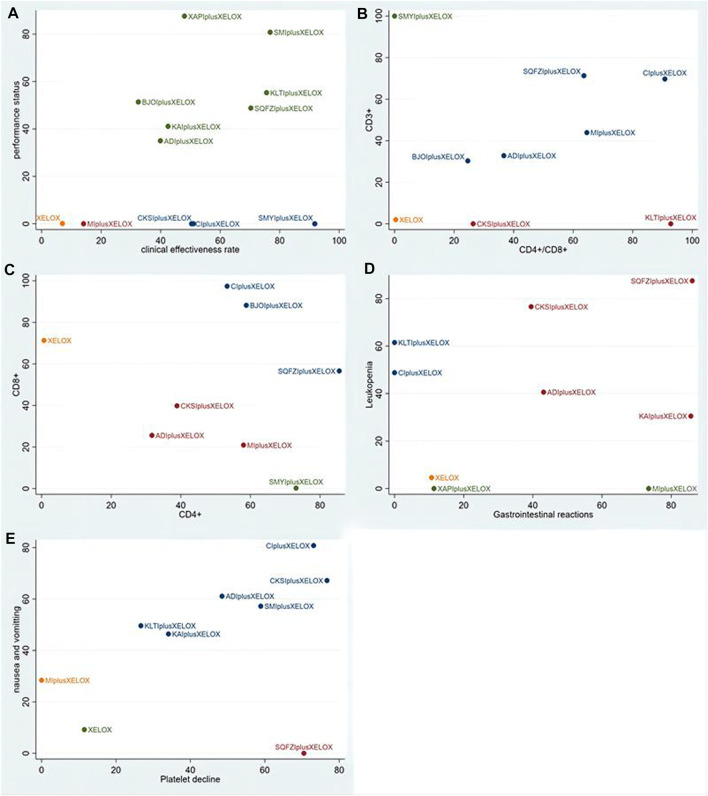
Cluster analysis plots: **(A)** the clinical effectiveness rate (*x*-axis) and performance status (*y*-axis); **(B)** CD4+/CD8+ (*x*-axis) and CD3^+^ (*y*-axis); **(C)** CD4^+^ (*x*-axis) and CD8+(*y*-axis); **(D)** Gastrointestinal reactions (*x*-axis) and Leukopenia (*y*-axis); **(E)** Platelet decline (*x*-axis) and nausea and vomitting (*y*-axis).

For primary outcome indicators, cluster analysis was used to evaluate the relative best treatment for CRC in this study. First, two-dimensional clustering results indicated that Shengmaiyin combined with XELOX, at the position furthestfrom the zero point, was the best in improving the clinical effectiveness. Xiaoaiping combined with XELOX was the best in improving the performance status. In contrast, the XELOX regimen alone had the worst comprehensive ranking of the examined regimens (Figure 6A). Second, Shengmaiyin combined with XELOX was the preferred treatment to increase the value of CD3^+^. Kanglaite combined with XELOX was thepreferred treatment to increase the rate of CD4+/CD8+. In addition, the XELOX regimen alone had the worstcomprehensive ranking of the examined regimens (Figure 6B). Third, Shenqifuzheng combined with XELOX was the best in increasing the value of CD4^+^. Cinobufacini combined with XELOX was the preferred treatment to increase the value of CD8+ (Figure 6C). Fourth, Shenqifuzheng combined with XELOX was not only the best in relieving Gastrointestinal reactions, but also the preferred treatment to relieve Leukopenia (Figure 6D).Fifth, Compound Kushen combined with XELOX was the best in relieving Platelet decline. Cinobufacini combined with XELOX was the preferred treatment to relieve nausea and vomitting (Figure 6E).

### Publication bias and sensitivity analysis

**FIGURE 7:**
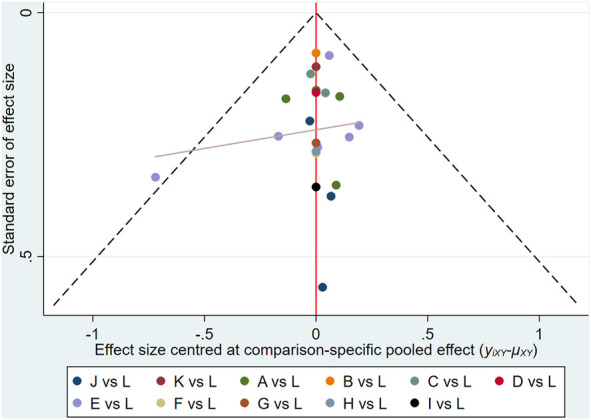
Funnel plots of outcomes: the clinical effectiveness rate: (A)ADIplusXELOX; (B)BJOIplus XELOX; (C)CIplusXELOX; (D)CKSIplusXELOX; (E)KAIplusXELOX; (F)KLTIplusXELOX; (G)MIplusXELOX; (H)SMIplusXELOX; (I)SMYIplusXELOX; (J)SQFZIplusXELOX; (K)XAPIplusXELOX; (L)XELOX.

STATA software was used to draw a comparison-adjusted funnel plot to evaluate publication bias based on the clinical effectiveness rate. As shown in Figure 7, the distribution of points in the funnel plot was visually asymmetrical around the midline, and the adjusted auxiliary line was almost non-perpendicular to the midline, suggesting the existence of publication bias among these studies. Moreover, sensitivity analysis was conducted by excluding each trial individually from the present study; the corresponding results of the current study were relatively robust.

## Discussion

The NMA approach was used to analyze the evidence from published RCTs and to compare the efficacy and safety of different CMIs. In summary, the results of the NMA performed in this study suggest that the XELOX regimen combined with Shengmaiyin, Shenmai, and Kanglaite injections has the more favorable clinical efficiency compared with the XELOX regimen alone; the XELOX regimen combined with Shengmaiyin, Shenqifuzheng, and Cinobufacini injections could improve the CD3^+^ values; the XELOX regimen combined with Shenqifuzheng, Shengmaiyin, and Brucea Javanica Oil injections could effectively raise the CD4^+^ values; the XELOX regimen combined with Cinobufacini and Brucea Javanica Oil injections could raise CD8^+^ values; the XELOX regimen combined with Kanglaite, Cinobufacini, and Matrine injections could improve the CD4+/CD8+ rates; and the XELOX regimen combined with Xiaoaiping, Shenmai, and Kanglaite injections improved the performance status. In addition, The use of Chinese medicine injections can effectively reduce the occurrence of adverse reactions. The XELOX regimen combined with Shenqifuzheng, Kangai, and Matrine injections could improve Gastrointestinal reactions; the XELOX regimen combined with Shenqifuzheng, Compound Kushen and Kanglaite injections could improve Leukopenia*;* the XELOX regimen combined with Compound Kushen, Cinobufacini and Shenqifuzheng injections could improve Platelet decline; the XELOX regimen combined with Cinobufacini, Compound Kushen and Aidi injections could improve nausea and vomitting*.* Statistically significant differences were observed between these groups.

The advantages of Shengmaiyin injection in improving the clinical efficiency and CD4^+^ and CD3^+^ values are obvious. Shengmaiyin is extracted from panax ginseng, ophiopogon japonicus, and schisandra chinensis. Modern pharmacological research has found that ginsenosides from ginseng have a two-way regulatory effect on patients’ humoral and cellular immunity, and have anti-tumor, anti-hepatotoxicity effects, etc. Ophiopogon japonicus can enhance myocardial tolerance to hypoxia and promote increased coronary blood flow; Schisandra chinensis has a significant function of protecting the cardiovascular system and improving microcirculation (Cui, 2016; Zhang et al., 2016; Huang et al., 2022). Some animal experiments have shown that Shengmaiyin injection can significantly reduce the content of NF-κB in tumor-bearing rats, suggesting that Shengmaiyin injection can inhibit the overexpression of TNF-instrument, IL-lβ, and NF-κB (Wang et al., 2011). Cinobufacini injection can effectively raise CD8^+^ and CD3^+^ values, improve the CD4+/CD8+ rate, and improve adverse reactions such as Platelet decline, nausea and vomiting. Cinobufacini is a water-soluble preparation produced by extracting and processing the whole skin of the Bufo gargarizans cantor, which is dried in the shade. The main function is to detoxify, reduce swelling, and relieve pain (Wu et al., 2015; Wu et al., 2021). It has been found that Cinobufacini has anti-tumor activity and enhances the effect of chemotherapy, and it can inhibit tumor growth and metastasis by inhibiting the expression of various growth factors. It has been widely used in the treatment of malignant tumors (Li, 2010; Yu et al., 2019). Relevant pharmacological studies have shown that the main components of Cinobufacini include toad venom lactones, indole alkaloids, peptides, cholesterol, etc. (Han, 2018). Based on the network pharmacology study, it was found that the active ingredients of Cinobufacini may act on the corresponding targets through the vascular endothelial growth factor signaling pathway, p53 signaling pathway, colorectal cancer signaling pathway, and tumor necrosis factor signaling pathway, and thus exert anti-tumor effects (Luo et al., 2021). Studies have shown that the combination of Cinobufacini with chemotherapy can effectively improve the clinical efficiency and performance status of the treatment, and can effectively relieve patients’ pain, and reduce the occurrence of Leukopenia, Platelet decline, and nausea and vomitting (Du, 2014). Kanglaite injection can effectively enhance the performance status and CD4+/CD8+ rate.In addition, Kanglaite injection can reduce the occurrence of Leukopenia. Kanglaite injection is an injectable emulsion of coix lacryma-jobi oil extracted from coix lacryma-jobi. It has the effect of benefiting qi and nourishing yin, supporting the essence, and it is rich in the natural active ingredient Coix lactone, with anti-tumor effects (Liang, 2017). Some studies have shown that Kanglaite can not only induce apoptosis and inhibit the proliferation of tumor cells by regulating the conduction pathway of P13K-Akt-mTOR or Fas/Fasl (Lu et al., 2008; Lu et al., 2009; Liu et al., 2014; Shi et al., 2017), but also inhibit and kill tumor cells by increasing the activity of T lymphocyte populations and enhancing the immune function of the body, so it is often used in combination with various chemotherapeutic drugs (Zhang et al., 2019), to improve patient tolerance, reduce adverse effects, relieve pain, and improve quality of life (Qi et al., 2010; Liu et al., 2019; Yu and Huang, 2019; Sun et al., 2020).

Currently, Chinese medical theory and treatment methods are being increasingly accepted, and TCM provides new ideas for the treatment of tumors. TCM has gradually shown its unique advantages in the treatment of tumors. Compared with traditional Chinese medicine tonics, CMIs have rapid efficacy, reliable action and bioavailability, and are widely used in the treatment of various diseases, becoming one of the most important directions in the development of modern Chinese medicine dosage forms (Duan et al., 2011). It was found that Shenmai, Shenqifuzheng, and Brucea Javanica Oil injections can enhance the immunity of tumor patients, improve hematopoietic function, reduce toxic side effects, and increase the treatment’s effectiveness (Qiao et al., 2001; Su et al., 2006; Liao and Xing, 2016). Shenmai injection is composed of panax ginseng and ophiopogon japonicus, which has the effect of benefiting qi and consolidating deficiencies, nourishing yin, and generating body fluid. It can improve the immunity of the body, improve the function of bone marrow, and significantly alleviate adverse reactions such as leukopenia, nausea, and vomiting caused by chemotherapy (Song and Xu, 2001; Zheng and Zhang, 2004). Shenqifuzheng injection is produced from codonopsis pilosula and astragalus mongholicus, which have the effects of benefiting Qi, promoting blood circulation, and resolving blood stasis. It contains saponin and astragalus methyloside, which can play a role in benefiting qi and tonifying the spleen, thus improving the body’s immune response and enhancing immunity (Hu, 2012). Animal experiments found that Shenqifuzheng injection combined with chemotherapy effectively improved cancer-caused fatigue in tumor-bearing nude mice (Yu et al., 2011). In addition, Shenqifuzheng injection can also improve Leukopenia and Platelet decline, and reduce the occurrence of gastrointestinal reactions, which has positive significance for tumor treatment (Meng, 2021). Brucea Javanica Oil injection is an extract from Chinese medicine Brucea Javanica; it is a non-cell-cycle-specific anti-tumor drug, mainly containing various fatty acids, such as oleic acid and linoleic acid, which have good affinity to cancer tissues (Jia et al., 2008). It was found that Brucea Javanica Oil injection could increase the activity of T cell subsets and NK cells and reduce COX-2 and PGE2 levels in tumor patients, showing significant anti-tumor effects in the clinical treatment of tumors (Lu et al., 2005).

At present, first-line chemotherapy regimens for colorectal cancer mainly include the FOLFOX regimen and XELOX regimen (Ducreux et al., 2011; Guo et al., 2016). The intervention evaluated in our study was the XELOX regimen; this restriction aimed to avoid potential interference caused by the different chemotherapeutic drugs in clinics. In addition, we found that reticulation metadata for CMIs combined with the FOLFOX regimen have been published, but no reticulation metadata were found for CMIs combined with the XELOX regimen. This study comprehensively retrieved the 11 types of CMIs widely used in clinical treatment and formulated strict inclusion criteria; this facilitated the analysis of which CMIs are most advantageous in the treatment of colorectal cancer. After research, in a comparison of CMIs combined with the XELOX regimen and CMIs combined with the FOLFOX regimen, the CMIs with better efficacy and safety were found to be different. In this study, the XELOX regimen combined with Shengmaiyin, Kanglaite, and Cinobufacini injections was associated with better clinical efficacy and safety compared with the XELOX regimen alone; However, in Ge’s study (Ge et al., 2016), the FOLFOX regimen combined with Shenqifuzheng, Aidi, and compound matrine injections was associated with better clinical efficacy and safety compared with the FOLFOX regimen alone. Therefore, for different chemotherapy regimens, the clinical application of CMIs needs to be considered more carefully.Nevertheless, the present NMA also had several limitations. First, CMIs are mainly used in China, and the included RCTs were performed in patients of Chinese descent; these factors all lead to geographical limitations. Therefore, it is unclear whether the conclusions of our study could be applied to populations in other geographic regions or other ethnic groups. Second, the reliability of our study was limited by the sample size, with a smaller sample size for inclusion in the study, especially for some types of CMIs. For Shenmai, Shengmaiyin, Xiaoaiping, Kanglaite, and Brucea Javanica Oil injections, in fact, only one clinical trial was included in the present study; therefore, further clinical or pharmacological research on the effects of different CMIs is necessary to support our findings. Third, the survival rate is an important indicator to judge the prognosis of tumor patients, but most studies did not report survival rates. Therefore, we suggest that clinical trials on patients with cancer should focus on this. Fourth, no direct head-to-head comparison was conducted between different CMIs, subgroup analysis and meta-regression were not done because of the non-uniformity of the data variables, so there are certain limitations. Fifth, because of the limitations of the regions and populations where CMIs are used, the literature included in this study was all in Chinese, and the overall quality was not high. Despite the above limitations, our network meta-analysis provides a complete assessment of patients with CRC and of different CMIs that can be used for CRC patients.

In this study, the adverse reactions of 9 injections were considered, and itwas found that Shenqifuzheng + XELOX, Kangai + XELOX and Matrine + XELOX couldeffectively relieve gastrointestinal reactions. Compound Kushen + XELOX, Cinobufacini + XELOX and Shenqifuzheng + XELOX could reduce the incidence of Platelet decline. Shenqifuzheng + XELOX, Compound Kushen + XELOX and Kanglaite + XELOX could reduce the incidence of Leukopenia. Cinobufacini + XELOX, Compound Kushen + XELOX and Aidi + XELOX could effectively relieve nausea and vomitting*.* It can be concluded that CMIs combined with the XELOX chemotherapy regimen has better safety, the adverse reactions of CMIs combined with the XELOX were significantly less frequent compared to those with XELOX chemotherapy alone, and CMIs can effectively improve the health of CRC patients, reduce toxicside effects, and improve the quality of life in clinical practice.

## Conclusion

In general, our NMA provides strong evidence supporting the use of different CMIs for CRC patients. Among different types of CMIs, the combination of Shengmaiyin, Kanglaite, or Cinobufacini injections and the XELOX regimen has significant treatment effects. In the future, RCTs that are better designed and larger, multi-center, head-to-head trials are needed to confirm these conclusions.

## Data Availability

The raw data supporting the conclusion of this article will be made available by the authors, without undue reservation.
