# Supplementary material for: Comparative efficacy and safety of Chinese medicine injections combined with capecitabine and oxaliplatin chemotherapies in treatment of colorectal cancer: A bayesian network meta-analysis
Source: Front Pharmacol. 2022 Nov 29;13:1004259. doi: 10.3389/fphar.2022.1004259 (PMC9745148; doi:10.3389/fphar.2022.1004259)
Supplement: Supplementary file 1 [file DataSheet1.PDF]

Supplementary Material

Content

Supplementary File 1 PRISMA checklist for network meta-analysis........2

Supplementary File 2 The Detailed Search Strategy........6

Supplementary Table1 Details of the included CMIs........14

Supplementary Figure1 Forest Plots of Outcomes........16

Supplementary File 3 English Editing Certificate........17

Supplementary File 1 PRISMA checklist for network meta-analysis

Section and Topic	Item #	Checklist item	Location where item is reported
**TITLE**			
Title	1	Identify the report as a systematic review.	P1
**ABSTRACT**			
Abstract	2	See the PRISMA 2020 for Abstracts checklist.	P1
**INTRODUCTION**			
Rationale	3	Describe the rationale for the review in the context of existing knowledge.	P2
Objectives	4	Provide an explicit statement of the objective(s) or question(s) the review addresses.	P2
**METHODS**			
Eligibility criteria	5	Specify the inclusion and exclusion criteria for the review and how studies were grouped for the syntheses.	P3
Information sources	6	Specify all databases, registers, websites, organisations, reference lists and other sources searched or consulted to identify studies. Specify the date when each source was last searched or consulted.	P3
Search strategy	7	Present the full search strategies for all databases, registers and websites, including any filters and limits used.	P2
Selection process	8	Specify the methods used to decide whether a study met the inclusion criteria of the review, including how many reviewers screened each record and each report retrieved, whether they worked independently, and if applicable, details of automation tools used in the process.	P3
Data collection process	9	Specify the methods used to collect data from reports, including how many reviewers collected data from each report, whether they worked independently, any processes for obtaining or confirming data from study investigators, and if applicable, details of automation tools used in the process.	P3

Section and Topic	Item #	Checklist item	Location where item is reported
Data items	10a	List and define all outcomes for which data were sought. Specify whether all results that were compatible with each outcome domain in each study were sought (e.g. for all measures, time points, analyses), and if not, the methods used to decide which results to collect.	P3
	10b	List and define all other variables for which data were sought (e.g. participant and intervention characteristics, funding sources). Describe any assumptions made about any missing or unclear information.	P3
Study risk of bias assessment	11	Specify the methods used to assess risk of bias in the included studies, including details of the tool(s) used, how many reviewers assessed each study and whether they worked independently, and if applicable, details of automation tools used in the process.	P4.9.10
Effect measures	12	Specify for each outcome the effect measure(s) (e.g. risk ratio, mean difference) used in the synthesis or presentation of results.	P3
Synthesis methods	13a	Describe the processes used to decide which studies were eligible for each synthesis (e.g. tabulating the study intervention characteristics and comparing against the planned groups for each synthesis (item #5)).	P6-7
	13b	Describe any methods required to prepare the data for presentation or synthesis, such as handling of missing summary statistics, or data conversions.	P5
	13c	Describe any methods used to tabulate or visually display results of individual studies and syntheses.	P6
	13d	Describe any methods used to synthesize results and provide a rationale for the choice(s). If meta-analysis was performed, describe the model(s), method(s) to identify the presence and extent of statistical heterogeneity, and software package(s) used.	P4
	13e	Describe any methods used to explore possible causes of heterogeneity among study results (e.g. subgroup analysis, meta-regression).	P3
	13f	Describe any sensitivity analyses conducted to assess robustness of the synthesized results.	P21
Reporting bias assessment	14	Describe any methods used to assess risk of bias due to missing results in a synthesis (arising from reporting biases).	P4
Certainty	15	Describe any methods used to assess certainty (or confidence) in the body of evidence for an outcome.	P4

Section and Topic	Item #	Checklist item	Location where item is reported
assessment			
**RESULTS**			
Study selection	16a	Describe the results of the search and selection process, from the number of records identified in the search to the number of studies included in the review, ideally using a flow diagram.	P5
	16b	Cite studies that might appear to meet the inclusion criteria, but which were excluded, and explain why they were excluded.	P3
Study characteristics	17	Cite each included study and present its characteristics.	P6-7
Risk of bias in studies	18	Present assessments of risk of bias for each included study.	P9-10
Results of individual studies	19	For all outcomes, present, for each study: (a) summary statistics for each group (where appropriate) and (b) an effect estimate and its precision (e.g. confidence/credible interval), ideally using structured tables or plots.	P15-17
Results of syntheses	20a	For each synthesis, briefly summarise the characteristics and risk of bias among contributing studies.	P4
	20b	Present results of all statistical syntheses conducted. If meta-analysis was done, present for each the summary estimate and its precision (e.g. confidence/credible interval) and measures of statistical heterogeneity. If comparing groups, describe the direction of the effect.	P10-14
	20c	Present results of all investigations of possible causes of heterogeneity among study results.	P9
	20d	Present results of all sensitivity analyses conducted to assess the robustness of the synthesized results.	P21
Reporting biases	21	Present assessments of risk of bias due to missing results (arising from reporting biases) for each synthesis assessed.	P4
Certainty of evidence	22	Present assessments of certainty (or confidence) in the body of evidence for each outcome assessed.	P9

Section and Topic	Item #	Checklist item	Location where item is reported
**DISCUSSION**			
Discussion	23a	Provide a general interpretation of the results in the context of other evidence.	P24
	23b	Discuss any limitations of the evidence included in the review.	P24-25
	23c	Discuss any limitations of the review processes used.	P25
	23d	Discuss implications of the results for practice, policy, and future research.	P26
**OTHER INFORMATION**			
Registration and protocol	24a	Provide registration information for the review, including register name and registration number, or state that the review was not registered.	P2
	24b	Indicate where the review protocol can be accessed, or state that a protocol was not prepared.	P26
	24c	Describe and explain any amendments to information provided at registration or in the protocol.	P26
Support	25	Describe sources of financial or non-financial support for the review, and the role of the funders or sponsors in the review.	P26
Competing interests	26	Declare any competing interests of review authors.	P26
Availability of data, code and other materials	27	Report which of the following are publicly available and where they can be found: template data collection forms; data extracted from included studies; data used for all analyses; analytic code; any other materials used in the review.	P26

Supplementary File 2 The Detailed Search Strategy

Search strategy of Pubmed：

No.	Search items
#1	"Colorectal Neoplasms"[MeSH Terms]
#2	"colorectal neoplasms"[Title/Abstract] OR "colorectal neoplasm"[Title/Abstract] OR ("neoplasm*"[All Fields] AND "Colorectal"[Title/Abstract]) OR "colorectal tumor*"[Title/Abstract] OR ("tumor*"[All Fields] AND "Colorectal"[Title/Abstract]) OR "colorectal cancer*"[Title/Abstract] OR ("cancer*"[All Fields] AND "Colorectal"[Title/Abstract]) OR "colorectal carcinoma*"[Title/Abstract] OR ("carcinoma*"[All Fields] AND "Colorectal"[Title/Abstract])
#3	#1 OR #2
#4	"Colonic Neoplasms"[MeSH Terms]
#5	"colonic neoplasms"[Title/Abstract] OR "colonic neoplasm"[Title/Abstract] OR ("neoplasm*"[All Fields] AND "Colonic"[Title/Abstract]) OR "colon neoplasm*"[Title/Abstract] OR ("neoplasm*"[All Fields] AND "Colon"[Title/Abstract]) OR "cancer of colon"[Title/Abstract] OR "colon cancer*"[Title/Abstract] OR ("cancer*"[All Fields] AND "Colon"[Title/Abstract]) OR "cancer of the colon"[Title/Abstract] OR "colonic cancer*"[Title/Abstract] OR ("cancer*"[All Fields] AND "Colonic"[Title/Abstract]) OR "colon adenocarcinoma*"[Title/Abstract] OR ("adenocarcinoma*"[All Fields] AND "Colon"[Title/Abstract])

#6	#4 OR #5
#7	"Rectal Neoplasms"[MeSH Terms]
#8	"rectal neoplasms"[Title/Abstract] OR ("neoplasm*"[All Fields] AND "Rectal" [Title/Abstract]) OR "neoplasm rectum"[Title/Abstract] OR "rectal neoplasm"[Title/Abstract] OR "rectum neoplasm*"[Title/Abstract] OR "rectal tumor*"[Title/Abstract] OR "tumor rectal"[Title/Abstract] OR "cancer of rectum"[Title/Abstract] OR "rectal cancer*"[Title/Abstract] OR "cancer rectal"[Title/Abstract] OR "rectum cancer*"[Title/Abstract] OR "cancer rectum"[Title/Abstract] OR "cancer of the rectum"[Title/Abstract]
#9	#7 OR #8
#10	#3 OR #6 OR #9
#11	"Injections"[MeSH Terms]
#12	"Injections"[Title/Abstract] OR "Injection"[Title/Abstract] OR "Injectables"[Title/Abstract] OR "Injectable"[Title/Abstract]
#13	#11 OR #12
#14	#3 OR #6 OR #9 AND #13
#15	"random*"[Title/Abstract] OR "randomized controlled trial"[Title/Abstract] OR "controlled clinical trial"[Title/Abstract] OR "double-blind"[Title/Abstract] OR "single-blind"[Title/Abstract] OR "Placebo"[Title/Abstract] OR "clinical trial*"[Title/Abstract] OR "RCT"[Title/Abstract] OR "RCTs"[Title/Abstract]
#16	#3 OR #6 OR #9 AND #13 AND #15

Search strategy of Embase：

No.	Search items
#1	'injection'/exp
#2	injections:ab,ti OR injection:ab,ti OR injectables:ab,ti OR injectable:ab,ti
#3	#1 OR #2
#4	'colorectal tumor'/exp
#5	colorectal tumor':ab,ti OR 'colorectal neoplasm':ab,ti OR 'neoplasm*, colorectal':ab,ti OR 'colorectal tumor*':ab,ti OR 'tumor*, colorectal':ab,ti OR 'colorectal cancer*':ab,ti OR 'cancer*, colorectal':ab,ti OR 'colorectal carcinoma*':ab,ti OR 'carcinoma*, colorectal':ab,ti OR 'colorectal neoplasms':ab,ti
#6	#4 OR #5
#7	'colon tumor'/exp
#8	colon tumor':ab,ti OR 'colonic neoplasms':ab,ti OR 'colonic neoplasm':ab,ti OR 'neoplasm*, colonic':ab,ti OR 'colon neoplasm*':ab,ti OR 'neoplasm*, colon':ab,ti OR 'cancer of colon':ab,ti OR 'colon cancer*':ab,ti OR 'cancer*, colon':ab,ti OR 'cancer of the colon':ab,ti OR 'colonic cancer*':ab,ti OR 'cancer*, colonic':ab,ti OR 'colon adenocarcinoma*':ab,ti OR 'adenocarcinoma*, colon':ab,ti
#9	#7 OR #8
#10	'rectum tumor'/exp
#11	rectum tumor':ab,ti OR 'rectal neoplasms':ab,ti OR 'neoplasm*, rectal':ab,ti OR 'neoplasm, rectum':ab,ti OR 'rectal neoplasm':ab,ti OR 'rectum neoplasm*':ab,ti OR 'rectal tumor*':ab,ti OR 'tumor, rectal':ab,ti OR 'cancer of rectum':ab,ti OR 'rectal cancer*':ab,ti OR 'cancer, rectal':ab,ti OR 'rectum cancer*':ab,ti OR

	'cancer, rectum':ab,ti OR 'cancer of the rectum':ab,ti
#12	#10 OR #11
#13	random*:ab,ti OR 'randomized controlled trial':ab,ti OR 'controlled clinical trial':ab,ti OR 'double blind':ab,ti OR 'single blind':ab,ti OR placebo:ab,ti OR 'clinical trial*':ab,ti OR rct:ab,ti OR rcts:ab,ti
#14	#3 AND #6 AND #9 AND #12 AND #13

Search strategy of Cochrane Library:

No.	Search items
#1	MeSH descriptor: [Colorectal Neoplasms] explode all trees
#2	(Colorectal Neoplasms):ti,ab,kw OR (Colorectal Neoplasm):ti,ab,kw OR (Neoplasm*, Colorectal):ti,ab,kw OR (Colorectal Tumor*):ti,ab,kw OR (Tumor*, Colorectal):ti,ab,kw
#3	(Colorectal Cancer*):ti,ab,kw OR (Cancer*, Colorectal):ti,ab,kw OR (Colorectal Carcinoma*):ti,ab,kw OR (Carcinoma*, Colorectal):ti,ab,kw
#4	#1 or #2 or #3
#5	MeSH descriptor: [Colonic Neoplasms] explode all trees
#6	(Colonic Neoplasms):ti,ab,kw OR (Colonic Neoplasm):ti,ab,kw OR (Neoplasm*, Colonic): ti,ab,kw OR (Colon Neoplasm*):ti,ab,kw OR (Neoplasm*, Colon):ti,ab,kw

#7	(Cancer of Colon):ti,ab,kw OR (Colon Cancer*):ti,ab,kw OR (Cancer*, Colon):ti,ab,kw OR (Cancer of the Colon):ti,ab,kw OR (Colonic Cancer*):ti,ab,kw
#8	(Cancer*, Colonic):ti,ab,kw OR (Colon Adenocarcinoma*):ti,ab,kw OR (Adenocarcinoma*, Colon):ti,ab,kw
#9	#5 or #6 or #7 or #8
#10	MeSH descriptor: [Rectal Neoplasms] explode all trees
#11	(Rectal Neoplasms):ti,ab,kw OR (Neoplasm*, Rectal):ti,ab,kw OR (Neoplasm, Rectum): ti,ab,kw OR (Rectal Neoplasm):ti,ab,kw OR (Rectum Neoplasm*):ti,ab,kw
#12	(Rectal Tumor*):ti,ab,kw OR (Tumor, Rectal):ti,ab,kw OR (Cancer of Rectum):ti,ab,kw OR (Rectal Cancer*):ti,ab,kw OR (Cancer, Rectal):ti,ab,kw
#13	(Rectum Cancer*):ti,ab,kw OR (Cancer, Rectum):ti,ab,kw OR (Cancer of the Rectum):ti,ab,kw
#14	#10 or #11 or #12 or #13
#15	MeSH descriptor: [Injections] explode all trees
#16	(Injections):ti,ab,kw OR (Injection):ti,ab,kw OR (Injectables):ti,ab,kw OR (Injectable):ti,ab,kw
#17	#15 or #16
#18	(Random*):ti,ab,kw OR (randomized controlled trial):ti,ab,kw OR (controlled clinical trial): ti,ab,kw OR (double-blind):ti,ab,kw OR (single-blind):ti,ab,kw
#19	(Placebo):ti,ab,kw OR (clinical trial*):ti,ab,kw OR (RCT):ti,ab,kw OR (RCTs):ti,ab,kw
#20	#18 or #19
#21	#4 and #9 and #14 and #17 and #20

Search strategy of Web of Science：

No.	Search items
#1	Colorectal Neoplasms (Topic) or Colorectal Neoplasm (Topic) or Neoplasm*, Colorectal (Topic) or Colorectal Tumor* (Topic) or Tumor*, Colorectal (Topic) or Colorectal Cancer* (Topic) or Cancer*, Colorectal (Topic) or Colorectal Carcinoma* (Topic) or Carcinoma*, Colorectal (Topic)
#2	Colonic Neoplasms (Topic) or Colonic Neoplasm (Topic) or Neoplasm*, Colonic (Topic) or Colon Neoplasm* (Topic) or Neoplasm*, Colon (Topic) or Cancer of Colon (Topic) or Colon Cancer* (Topic) or Cancer*, Colon (Topic) or Cancer of the Colon (Topic) or Colonic Cancer* (Topic) or Cancer*, Colonic (Topic) or Colon Adenocarcinoma* (Topic) or Adenocarcinoma*, Colon (Topic)
#3	Rectal Neoplasms (Topic) or Neoplasm*, Rectal (Topic) or Neoplasm, Rectum (Topic) or Rectal Neoplasm (Topic) or Rectum Neoplasm* (Topic) or Rectal Tumor* (Topic) or Tumor, Rectal (Topic) or Cancer of Rectum (Topic) or Rectal Cancer* (Topic) or Cancer, Rectal (Topic) or Rectum Cancer* (Topic) or Cancer, Rectum (Topic) or Cancer of the Rectum (Topic)
#4	Injections (Topic) or Injection (Topic) or Injectables (Topic) or Injectable (Topic)
#5	Random* (Topic) or randomized controlled trial (Topic) or controlled clinical trial (Topic) or double-blind (Topic) or single-blind (Topic) or Placebo (Topic) or clinical trial* (Topic) or RCT (Topic) or RCTs (Topic)
#6	#127 AND #122 AND #117 AND #111 AND #106

Search strategy of China National Knowledge Infrastructure

No.	Search items
#1	SU %='中药注射液'
#2	SU %= '直肠癌’ OR SU %= '大肠癌' OR SU %= '结直肠癌' OR SU %= '结肠癌'
#3	FT = '随机'
#4	#1 AND #2 AND #3

Search strategy of Wanfang Database

No.	Search items
#1	主题:(中药注射液)
#2	主题:(直肠癌) or 主题:(大肠癌) or 主题:(结直肠癌) or 主题:(结肠癌)
#3	全部:(随机)
#4	#1 AND #2 AND #3

Search strategy of Chinese Biomedical Literature Database

No.	Search items
#1	"中药注射液"[常用字段:智能]
#2	"直肠癌"[常用字段:智能] OR "大肠癌"[常用字段:智能] OR "结直肠癌"[常用字段:智能] OR "结肠癌"[常用字段:智能]
#3	"随机"[全部字段:智能]
#4	#1 AND #2 AND #3

Search strategy of Weipu Journal Database

No.	Search items
#1	M=(中药注射液)
#2	M=(直肠癌 OR 大肠癌 OR 结直肠癌 OR 结肠癌)
#3	U=(随机)
#4	#1 AND #2 AND #3

Supplementary Table 1 Details of the included CMIs

Chinese medicine injections	Species	Raw material of botanical drugs
Shengmaiyin injection	Panax ginseng;Ophiopogon japonicus; Schisandra chinensis	Panax ginseng (C.A.Mey.)[Araliaceae;Panax ginseng dry leaf with stem]; Ophiopogon japonicus (Thunb.) Ker Gawl.[Asparagaceae;Ophiopogon japonicus dwarf lily-turf];Schisandra chinensis (Turcz.) Baill.[Schisandraceae;Schisandra chinensis fruit]
Shenmai injection	Panax ginseng;Ophiopogon japonicus	Panax ginseng (C.A.Mey.)[Araliaceae;Panax ginseng dry leaf with stem]; Ophiopogon japonicus (Thunb.) Ker Gawl.[Asparagaceae;Ophiopogon japonicus dwarf lily-turf]
Kanglaite injection	Coix lacryma-jobi	Coix lacryma-jobi L. [Poaceae；Coix lacryma-jobi seed]
Shenqifuzheng injection	Codonopsis pilosula; Astragalus mongholicus Bunge	Codonopsis pilosula (Franch.) Nannf.[Campanulaceae;Codonopsis pilosula root];Astragalus mongholicus Bunge [Fabaceae;Astragalus mongholicus root]
Cinobufacini injection	——	——
Brucea Javanica Oil injection	Brucea javanica	Brucea javanica (L.) Merr. [Simaroubaceae;Brucea javanica seed]
Matrine injection	Sophora flavescens	Sophora flavescens Aiton [Fabaceae;Sophora flavescens root]
Xiaoaiping injection	Marsdenia tenacissima	Marsdenia tenacissima (Roxb.) Moon [Apocynaceae；Marsdenia tenacissima stem]
Aidi injection	Panax ginseng; Astragalus mongholicus Bunge；	Panax ginseng (C.A.Mey.)[Araliaceae;Panax ginseng dry leaf with stem];Astragalus mongholicus Bunge [Fabaceae;Astragalus mongholicus root]；Eleutherococcus senticosus (Rupr. & Maxim.) Maxim. [Araliaceae；

	Eleutherococcus senticosus	radix and rhizome]
Kangai injection	Astragalus mongholicus Bunge;Panax ginseng;Sophora flavescens	Astragalus mongholicus Bunge [Fabaceae;Astragalus mongholicus root];<br>Panax ginseng (C.A.Mey.)[Araliaceae;Panax ginseng dry leaf with stem];<br>Sophora flavescens Aiton [Fabaceae;Sophora flavescens root]
Compound Kushen injection	Sophora flavescens ;Smilax glabra	Sophora flavescens Aiton [Fabaceae;Sophora flavescens root];<br>Smilax glabra Roxb. [Smilacaceae;Smilax glabra rhizome]

Supplementary Figure 1 Forest Plots of Outcomes

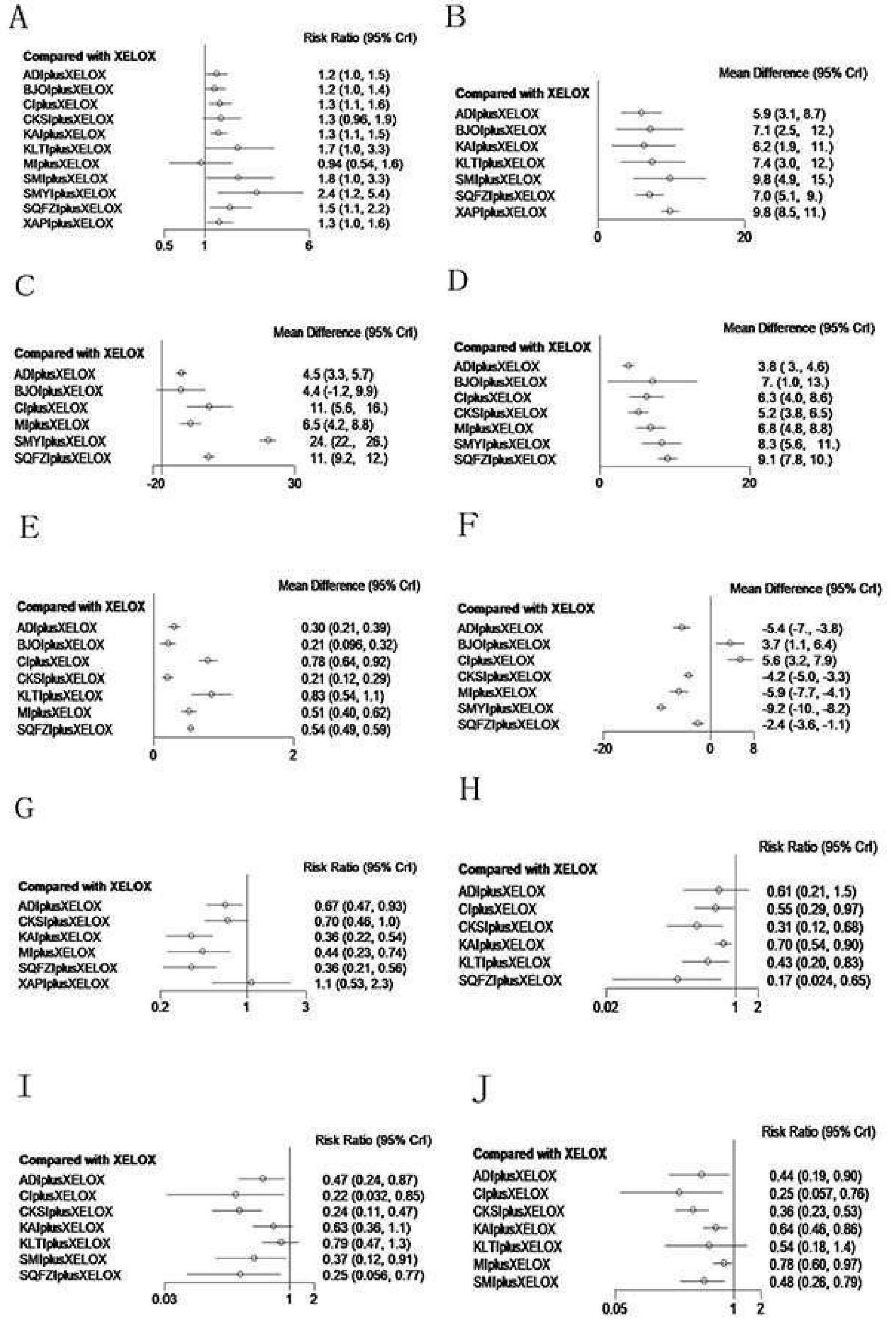

SMYI, Shengmaiyin injection; SMI, Shenmai injection; KLT, Kanglaite injection; SQFZI, Shenqifuzheng injection; CI, Cinobufacini injection; BJOI, Brucea Javanica Oil injection; MI,Matrine injection; XAPI, Xiaoaiping injection; ADI, Aidi injection; KAI, Kangai injection; CKSI, Compound Kushen injection. (A) Clinical effectiveness rate; (B) KPS; (C) CD3+; (D) CD4+; (E) CD4+/CD8+; (F) CD8+; (G) Gastrointestinal reactions; (H) Leukopenia; (I) Platelet decline; (J) nausea and vomitting. 95% CrI: 95% credible interval.

Supplementary File 3 English Editing Certificate

We certify that the following article

Comparative Efficacy and Safety of Chinese Herbal Injections Combined With Capecitabine and Oxaliplatin Chemotherapies in Treatment of Colorectal Cancer: A Bayesian Network Meta-Analysis

kun Zhang

has undergone English language editing by MDPI. The text has been checked for correct use of grammar and common technical terms, and edited to a level suitable for reporting research in a scholarly journal.
MDPI uses experienced, native English speaking editors. Full details of the editing service can be found at
► https://www.mdpi.com/authors/english.

MDPI Multidisciplinary Digital Publishing Institute

Basel, Switzerland
July 2022

AUTHORservices
